# An aptamer‐guided fluorescence polarisation platform for extracellular vesicle liquid biopsy

**DOI:** 10.1002/jev2.12502

**Published:** 2024-09-02

**Authors:** Cuong Viet Pham, Rocky Chowdhury, Shweta Patel, Satendra Kumar Jaysawal, Yingchu Hou, Huo Xu, Lee Jia, Yu‐mei Zhang, Xiaowei Wang, Wei Duan, Dongxi Xiang

**Affiliations:** ^1^ School of Medicine Deakin University Waurn Ponds VIC Australia; ^2^ Molecular Imaging and Theranostics Laboratory Baker Heart and Diabetes Institute Melbourne VIC Australia; ^3^ Laboratory of Tumor Molecular and Cellular Biology College of Life Sciences Shaanxi Normal University Xi'an Shaanxi China; ^4^ College of Materials and Chemical Engineering Minjiang University Fuzhou Fujian China; ^5^ Department of Cardiometabolic Health University of Melbourne VIC Australia; ^6^ State Key Laboratory of Systems Medicine for Cancer, Shanghai Cancer Institute Shanghai Jiaotong University Shanghai China; ^7^ Department of Biliary‐Pancreatic Surgery the Renji Hospital Affiliated to Shanghai Jiaotong University School of Medicine Shanghai China

**Keywords:** aptamer, cancer, chemical antibody, exosome, extracellular vesicles, fluorescence polarisation, liquid biopsy, tetraspanin

## Abstract

The translation of discoveries on extracellular vesicle (EV) based cancer biomarkers to personalised precision oncology requires the development of robust, sensitive and specific assays that are amenable to adoption in the clinical laboratory. Whilst a variety of elegant approaches for EV liquid biopsy have been developed, most of them remain as research prototypes due to the requirement of a high level of microfabrication and/or sophisticated instruments. Hence, this study is set to develop a simple DNA aptamer‐enabled and fluorescence polarisation‐based homogenous assay that eliminates the need to separate unbound detection ligands from the bound species for EV detection. High specificity is achieved by immobilising EVs with one set of antibodies and subsequently detecting them with a DNA aptamer targeting a distinct EV biomarker. This two‐pronged strategy ensures the removal of most, if not all, non‐EV substances in the input biofluids, including soluble proteins, protein aggregates or non‐vesicular particles, prior to quantifying biomarker‐positive EVs. A limit of detection of 5.0 × 10^6^ EVs/mL was achieved with a linear quantification range of 5.0 × 10^8^ to 2.0 × 10^10^ EVs/mL. Facilitated by a multiple parametric analysis strategy, this aptamer‐guided fluorescence polarisation assay was capable of distinguishing EVs from three different types of solid cancer cells based on quantitative differences in the levels of the same sets of biomarkers on EVs. Given the simplicity of the method and its ease of implementation in automated clinical biochemistry analysers, this assay could be exploited for future EV‐based continuous and real‐time monitoring of the emergence of new macro‐ or micro‐metastasis, cancer progression as well as the response to treatment throughout different stages of cancer management in the clinic.

## 1 INTRODUCTION

Currently, lab‐on‐a‐chip‐based devices, nanoparticle tracking analysis (NTA), high‐resolution single EV flow cytometry and enzyme‐linked immunosorbent assay (ELISA) are the main approaches adopted by EV researchers to detect and quantify extracellular vesicles in a high‐throughput manner. However, lab‐on‐a‐chip technologies such as microfluidics and micromachined chips require high‐level microfabrication techniques and biomedical engineering, as well as sophisticated instruments to read signals and analyse data (Chen et al., 2021; Sugumar & Kong, 2008; Wang et al., 2020), making these technologies difficult to be widely employed in general EV research laboratories and clinical diagnostic settings (Leggio et al., 2023). NTA suffers from several limitations including low labelling capacity in fluorescence mode, the requirement of relatively high sample volume (Thane et al., 2019), low sensitivity and underestimating EV concentration. Recently, high‐sensitivity flow cytometry has been utilised not only for the detection and quantification of EVs but also for the characterisation of a single EV with the potential of high‐throughput and multiplex analysis. However, swarm effects, high background from biological samples, limitation in the sizes of detected particles and the high cost of the instrument are hindering its wide adoption by EV researchers (Botha et al., 2021; Deville et al., 2021; Russell et al., 2019). Whilst ELISA is a commonly used analytical biochemical assay, the limit of detection of ELISA in measuring EV concentration falls into the scale of 10^9^‒10^10^ EVs/mL (López‐Cobo et al., 2018; Martín‐Gracia et al., 2020). Therefore, a sensitive and simple method, that is, capable of specifically detecting and quantifying EVs is urgently needed for both research and clinical applications.

Fluorescence polarisation (FP) has been utilised in high‐throughput assays in drug discovery as well as in clinical settings for the past few decades following Weber's pioneering work (Jameson & Ross, 2010). The FP operates as a homogenous assay with several distinct advantages, including good reproducibility, high sensitivity, a high degree of autonomy, adaptability to work with low volumes (∼10 µL) and no requirement for the separation of free ligand from bound one (Hall et al., 2016; Jameson & Ross, 2010; Moerke, 2009). In contrast, the heterogenous techniques require the separation of unbound and bound species, and are thus not only more laborious but also inaccurate in quantification of real biomolecular interactions in solution (Corporation, 2006). Moreover, FP is a ratiometric method which is insensitive to interference and/or artifacts derived from inner filter effects, photobleaching as well as the change in environmental conditions. FP is also generally independent of the absorbance or colour quenching from library compounds (Corporation, 2006; Glickman et al., 2002; Hall et al., 2016; Lakowicz, 2006; Zhang et al., 2019). Furthermore, some other popular fluorescence‐based homogeneous assays, such as fluorescence resonance energy transfer (FRET) and time‐resolved FRET (TR‐FRET), require multiple labels to be conjugated to the probe, whilst a single fluorophore label is needed in FP assay (Corporation, 2006; Lakowicz, 2006). As reflected light and light scattering are partly or totally polarised light (Jameson & Ross, 2010), large vesicles with size larger than wavelength of incident light are not suitable subjects for FP assays because they can reflect and scatter incident light in high intensity, thus observed light in high polarisation degree may mask the specific FP from ligand‐vesicle interaction (Chýlek, 1986; Yada, 2018). Fortunately, the dimension of small extracellular vesicles (EVs < 200 nm) is smaller than the excitation wavelength of common fluorophores (400‒700 nm) (Hall et al., 2016), generating no reflected light and dim scattered light in FP assays (Chýlek, 1986; Yada, 2018). Most critically, the high volume and mass of extracellular nanovesicles (>700 kDa [Corso et al., 2017]) together make these small EVs ideal analytes for FP‐based detection as long as small size detection ligands, such as aptamers, are employed (Panagopoulou et al., 2020).

To achieve optimal performance and high sensitivity with a FP assay, a robust fluorescent probe with a molecular mass smaller than 15 kDa is desirable, as such a small‐sized probe ensures a high FP as a free probe but a very low FP upon binding to its target (Pope et al., 1999; Yao et al., 2020). Although the commonly used lipophilic dyes in the EV field, such as PKH family and the carbocyanine dyes (DiI, DiO), are very small ligands (∼1 kDa), however, they are not ideal FP probes for EVs due to their intrinsic limitations such as non‐specific binding to non‐EV particles and artefactual results from dye aggregations (Melling et al., 2022). In contrast, aptamers, also known as chemical antibodies, are single‐stranded nucleic acids that fold into a defined three‐dimensional structure and thus able to interact with their targets specifically (Dunn et al., 2017). Being a class of small but specific affinity probes with a molecular mass generally small than 15 kDa, aptamers are ideal probes for detecting EVs through FP‐based assays.

In this study, a method of fluorescence polarisation using aptamers for the detection of extracellular nanovesicles (FluoPADE) is developed and applied to a variety of EV samples. FluoPADE assay is based on the high specificity of antibodies to immobilise EVs followed by the use of small‐sized aptamers to efficiently and quantitatively detect and classify cancer cell line‐derived EVs from cell culture media as well as in human plasma.

## 2 MATERIALS AND METHODS

### 2.1 Antibodies

The sources of antibodies used in this study are as following: anti‐human CD9 APC‐conjugated antibody (Thermo Fisher Scientific, Cat. No.: A15698), anti‐human CD9 biotin‐conjugated antibody (BioLegend, Cat. No.: 312112), anti‐human CD9 PE‐conjugated antibody (BioLegend, Cat. No.: 312106), anti‐human CD81 APC‐conjugated antibody (Thermo Fisher Scientific, Cat. No.: 17‐0819‐42), anti‐human CD81 biotin‐conjugated antibody (BioLegend, Cat. No.: 349514), anti‐human CD81 FITC‐conjugated antibody (BioLegend, Cat. No.: 349504), anti‐human CD63 APC‐conjugated antibody (Thermo Fisher Scientific, Cat. No.: A15712), anti‐human CD63 biotin‐conjugated antibody (BioLegend, Cat. No.: 353017), anti‐human CD63 FITC‐conjugated antibody (BioLegend, Cat. No.: 353006), anti‐human EpCAM Alexa Fluor® 647‐conjugated antibody (R&D Systems, Cat. No.: FAB9601R100UG), anti‐human EpCAM biotin‐conjugated antibody (BioLegend, Cat. No.: 324216), anti‐human HER2 FITC‐conjugated antibody (BioLegend, Cat. No.: 324404), anti‐human HER2 PE‐conjugated antibody (BioLegend, Cat. No.: 324405), anti‐human HER2 APC‐conjugated antibody (BioLegend, Cat. No.: 324408) and mouse IgG1 biotin‐conjugated isotype control (BioLegend, Cat. No.: 400104).

### 2.2 Aptamers

All the FITC‐conjugated DNA aptamers involved in this study were synthesised by Integrated DNA Technologies, Inc., Coralville, USA. The sequences for the full‐length of DNA aptamers used are shown in Table 1.

**TABLE 1:** Sequences of aptamers used in this study.

Aptamer	Respective biomarker	Sequences (5′→3′)	Reference
FITC‐CD63‐BP	CD63	5′‐FITC‐CAC CCC ACC TCG CTC CCG TGA CAC TAA TGC TA‐idT‐3′	Jiang et al. (2017)
FITC‐HER2‐HApt	HER2	5′‐FITC‐ GCA GCG GTG TGG GGG CAG CGG TGT GGG GGC AGC GGT GTG GGG‐idT‐3′	Lee et al. (2015)
FITC‐negative control aptamer	Annexin A1 (cytosolic face of the plasma membrane)	5′‐FITC‐GCG CGA ATG GGC GCG C ‐idT‐3′	Bavi et al. (2020)

*Note*: Fluorescein isothiocyanate (excitation peak of 491 nm and emission peak of 516 nm).

Abbreviations: FITC, fluorescein isothiocyanate; idT, inverted deoxythymidine.

### 2.3 Cell culture

HEK‐293 (human embryonic kidney 293) cell line was obtained from Prof. Alister Ward, School of Medicine, Deakin University. SKBR3 (human breast cancer) cell line was a gift from Dr. Belinda Parker at La Trobe University. HT‐29 (human colorectal adenocarcinoma, ATCC^®^ HTB38™), MDA‐MB‐231 (human breast cancer, ATCC^®^ HTB‐26™) and HepG2 (human hepatocarcinoma, ATCC® HB‐8065™) cell lines were purchased from American Type Culture Collection (ATCC, Manassas, VA). HER2 gene knockout MDA‐MB‐231 (HER2‐KO MDA‐MB‐231, clone MG57) cell line was produced in collaboration with Monash Genome Modification Platform (Monash University, Melbourne, Australia). All the above cells were cultured in Dulbecco's Modified Eagle's Medium (DMEM, Gibco) supplemented with 10% foetal bovine serum (FBS, Gibco) and 1X Glutamax (Gibco) in a humidified atmosphere containing 5% CO_2_ at 37°C. For sub‐culturing cells from monolayer, cell culture medium was removed, and cells were rinsed gently with sterilised phosphate saline buffer (PBS) twice, followed by detachment with 0.05% trypsin‐EDTA (Sigma) at 37°C until cells were rounded up and detached from the surface of flasks. Then, complete medium was added to the flasks to inactivate the trypsin. Cells were centrifuged at 400 × *g* for 5 min and collected at room temperature (RT), followed by resuspension with appropriate buffer or medium.

### 2.4 Preparation of human plasma

Whole blood samples in EDTA tubes were obtained from the Australian Red Cross Lifeblood (Melbourne, Australia). The blood was then centrifuged at 1200 × *g* for 10 min at 4°C (Eppendorf, 5810R), and plasma was transferred to a clean Eppendorf tube and centrifuged again at 1800 × *g* for 10 min at 4°C before it was aliquoted and stored at −80°C until use (Lobb et al., 2015).

### 2.5 Production of cell‐derived extracellular vesicles

At ∼80% confluence, cells were washed five times with sterile 0.22‐µm‐filtered PBS (F‐PBS) and the medium for culturing was changed to DMEM containing 1Χ Glutamax® without bovine serum. The cells were then incubated for 48 h at 37°C with 5% CO_2_ and saturated humidity. Next, the conditioned medium containing extracellular vesicles was collected and further sequentially centrifuged at 400 *× g* for 10 min followed by 2000 *× g* for 20 min in a swinging bucket rotor (S‐4‐72, Eppendorf) and finally at 10,000 *× g* for 30 min in a fixed angle rotor (FA‐45‐6‐30, Eppendorf) at 4°C using Centrifuge 5810 R (Eppendorf). The clarified conditioned cell culture supernatant was used for EV isolation (Théry et al., 2006).

### 2.6 Isolation of cell‐derived extracellular vesicles

#### 2.6.1 Ultrafiltration

The 10,000 × *g* conditioned cell culture supernatant was concentrated at 3500 × *g* for 30 min using Centricon® Plus‐70 Centrifugal Filter Units, MWCO 10 kDa (Merck, Cat. No.: UFC701008). The concentrated EVs mixture was used freshly or stored at −80°C for up to 2 months.

#### 2.6.2 Ultracentrifugation

The 10,000 × *g* conditioned cell culture medium was ultracentrifugated at 100,000 × *g* at 4°C for 90 min using a TLS‐55 rotor. The supernatant was discarded, and the pellet was resuspended with 1 mL of cold F‐PBS followed by a second ultracentrifugation at 100,000 × *g* at 4°C for 90 min. After the removal of the supernatant, the pellet was resuspended in 100 µL of F‐PBS and stored at –80°C up to 2 months for further use.

### 2.7 Fluorescence polarisation using aptamers for the detection of extracellular nanovesicles (FluoPADE assays)

Streptavidin‐coated wells (Thermo Fisher Scientific, Cat. No.: 15503) were washed with 200 µL of 0.22 µm filtered PBS plus 0.1% BSA and 0.05% Tween 20 (Washing buffer‐A) twice. Then, 100 µL of 8 µg/mL biotinylated capture antibody (either anti‐EpCAM antibody or anti‐CD9/CD81 antibody, 1:1. w/w) in Washing buffer‐A was added into each well. The well was incubated for 30 min at room temperature on a shaker (Thermoline Scientific, Model No.: TL400) at 120 rpm before being washed twice with 200 µL of Washing buffer‐A and once with 200 µL of F‐PBS. Next, 100 µL of cell culture‐derived EVs diluted with either 0.22‐µm‐membrane‐filtered PBS or human plasma (90 µL of human plasma) to desirable EV concentration was incubated with the well‐coated with the indicated antibody at 4°C overnight. The supernatant containing non‐immobilised EVs, and other components were discarded and the well with immobilised EVs was washed with 200 µL of 0.22‐µm‐membrane‐filtered PBS plus 0.1% Tween 20 (Washing buffer‐B) for three times. After that, 100 µL of 5.0 nM FITC‐conjugated CD63 aptamer (CD63‐BP) or FITC‐conjugated HER2 aptamer (HER2‐HApt) was introduced and incubated with the well for 1 h in the dark on a shaker (Thermoline Scientific, Model No.: TL400) at 120 rpm at room temperature. Finally, FP signals were recorded using a multilabel plate reader CLARIOstar Plus (BMG Labtech). An excitation filter at 485 nm and an emission filter at 535 nm were used for FP measurement. FP value of each sample was the mean value of three different wells, whilst the FP value of each well was the mean signal from three different measurements on that well.

The difference in fluorescence polarisation between samples with and without immobilised EVs or the change in fluorescence polarisation (ΔFP) was calculated using the following equation: $\Delta FP=FP_{samplewithEVs}-FP_{samplewithoutEVs}$ and expressed in millipolarisation (mP) units, where $FP_{\mathrm{samp}\mathrm{lewi}\mathrm{thEVs}}$ is a FP value of sample containing cancer cell‐derived EVs, $FP_{samplewithoutEVs}$ is a FP value of FITC‐labelled ligand in capture antibody‐coated well in the absence of cancer cell‐derived EVs. For EV samples spiked into human plasma, $FP_{samplewithoutEVs}$ is the FP value of the sample treated with human plasma without cancer cell‐derived EVs.

For assays to determine selectivity and specificity, control samples with EVs immobilised in the microwells were first treated with Triton X‐100 at the concentration of 1% for 30 min at room temperature to lyse EVs. The control of free EpCAM protein was established by the addition of 100 µL of 50 nM free EpCAM protein (Sino Biological, Cat. No.: 10694‐H08H). Other controls with EpCAM‐negative or HER2‐negative EVs were prepared using the same concentration of maker‐positive EVs as the experimental groups.

### 2.8 Determination of sensitivity of FluoPADE assays

A mixture of cancer marker (EpCAM or HER2)‐positive and marker‐negative EVs were prepared at various ratios whilst the total EV concentration remained constant. The lowest concentration of cancer marker‐positive EVs was set at the limit of detection (LOD) of the corresponding FluoPADE assay. In FluoPADE assay using CD63‐BP aptamer (EVs were captured using EpCAM antibody), the mixtures of HT‐29 EVs (EpCAM‐positive EVs) and HEK‐293 EVs (EpCAM‐negative EVs) were prepared at five different ratios of 1:2000, 1:1000, 1:500, 1:100 and 1:10 with the total concentration of EVs maintained at 1.0005 × 10^10^ EVs/mL. In the FluoPADE assay using HER2‐HApt aptamer (EVs were captured using CD9/CD81 antibodies), the pools of EVs from HER‐2‐positive SKBR3 cells and EVs from HER2 gene knockout MDA‐MB‐231 cells were prepared at ratios of 1:1000, 1:750, 1:500, 1:100 and 1:10, with a constant total EV concentration of 3.0030 × 10^10^ EVs/mL. Upon the addition of the detection ligand, FP signals were measured as described in Section 2.5. The sensitivity of a given EV detection assay was determined as the ratio at which the corresponding signal (ΔFP) was equal or higher than the sum of the respective blank signal and three times the standard deviation of the blank signal ($Mean_{blank}+3\times SD_{blank}$).

### 2.9 Distinguishing EVs derived from different cancer cell lines based on the different levels of the surface marker proteins on EV using FluoPADE assays

In this assay, EVs from three different cancer cell lines were distinguished or separated on a 2‐D plot based on the same surface marker proteins expressed at different levels on EVs. Briefly, EVs derived from three different cancer cell lines, that is, HT‐29, SKBR3 and HepG2, were spiked into human plasma, in which 10 µL of 1.0 × 10^8^ EVs from each cancer cell line was spiked into 90 µL of 9.0 × 10^8^ human plasma EVs from each of the six different donors, resulting in a final concentration of total EVs of 1.0 × 10^10^ EVs/mL and a ratio of cancer cell line EV versus plasma EV of 1:9 (v/v). Next, FluoPADE assays were performed using either anti‐EpCAM antibody to capture EVs and CD63‐BP aptamer as a detection ligand or a mixture of anti‐CD9/CD81 antibody (1:1, w/w) to capture EVs and HER2‐HApt aptamer as a detection ligand. The ΔFP for HER2/CD9‐CD81 biomarkers in the six samples was then plotted in the Y‐axis whereas the ΔFP for CD63/EpCAM biomarkers in the six samples was plotted in the X‐axis in the 2‐dimensional chart for separating clusters of EVs based on their origin.

### 2.10 Fluorescence intensity‐based detection of cancer‐derived EVs

Various concentrations of input HT‐29 EVs or SKBR3 EVs were prepared in F‐PBS or human plasma, at a ratio of 10 µL of EVs versus 90 µL of F‐PBS or plasma. The EVs were immobilised by anti‐EpCAM antibody for detection using either CD63‐BP aptamer or CD63 antibody. Alternatively, EVs were immobilised by a mixture of anti‐CD9/CD81 antibodies for the detection using HER2‐HApt aptamer or HER2 antibody. The EV capture was performed in the same way as described in Section 2.7. The immobilised EVs, then, were incubated with 100 µL of either 800 nM aptamer or 50 nM of antibody for 1 h in the dark on a shaker (Thermoline Scientific, Model No.: TL400) at 120 rpm at room temperature. After three washes with 200 µL of Washing buffer‐A, the fluorescence intensity (FI) of the well was measured using a multilabel plate reader CLARIOstar Plus (BMG Labtech). An excitation filter at 485 nm and an emission filter at 535 nm were used.

The fluorescence intensity was calculated with the following equation:

$$FI=FI_{sample\;with\;EVs}-FI_{sample\;without\;EVs}$$

where $FI_{samplewithEVs}$ is the FI value of a sample containing cancer cell‐derived EVs, $FI_{samplewithoutEVs}$ is the FI value of the control well containing FITC‐labelled ligand in the absence of cancer cell‐derived EVs. For EV samples spiked into human plasma, $FI_{samplewithoutEVs}$ is the FI value of the sample treated with human plasma without cancer cell‐derived EVs.

### 2.11 Data analysis

All results are shown as means ± standard deviation (means ± SD) from triplicates unless otherwise stated. The statistical analyses, calibration curves, fitting equations and coefficients of determination (*R*‐squared) were performed using GraphPad Prism 8.0. The one‐way analysis of variance (ANOVA) was used for comparisons of more than two groups and the differences between two specific groups were analysed by *t*‐test. In all tests, *p* ≤ 0.05 (*), *p* ≤ 0.01 (**), *p* ≤ 0.001 (***) and *p* ≤ 0.0001 (****) were considered as statistically significant, whereas *p* > 0.05 (ns) was considered as not statistically significant.

Calibration curves in FluoPADE assays and binding curves in *K_D_* determination assays were fitted using non‐linear regression for specific binding ($Y=\;\frac{A\times X}{B+X})$. Calibration curves, fitting equations and coefficients of determination (*R*‐squared) in other assays were determined using linear regression model (𝑌 = 𝑎 × 𝑋 + 𝑏). The limit of detection (LOD) is the EV concentration at which the signal is equal to or higher than the sum of the respective blank signal and three times the standard deviation of the blank signal ($Mean_{blank}$ +3 × $SD_{blank}$). The limit of quantification (LOQ) is the EV concentration at which the signal is equal to the sum of the respective blank signal plus 10 times the standard deviation of the blank signal ($Mean_{blank}$ +10 × $SD_{blank}$) or 3.3 × LOD.

## 3 RESULTS

### 3.1 Assay design and experimental strategies

**SCHEME 1:**
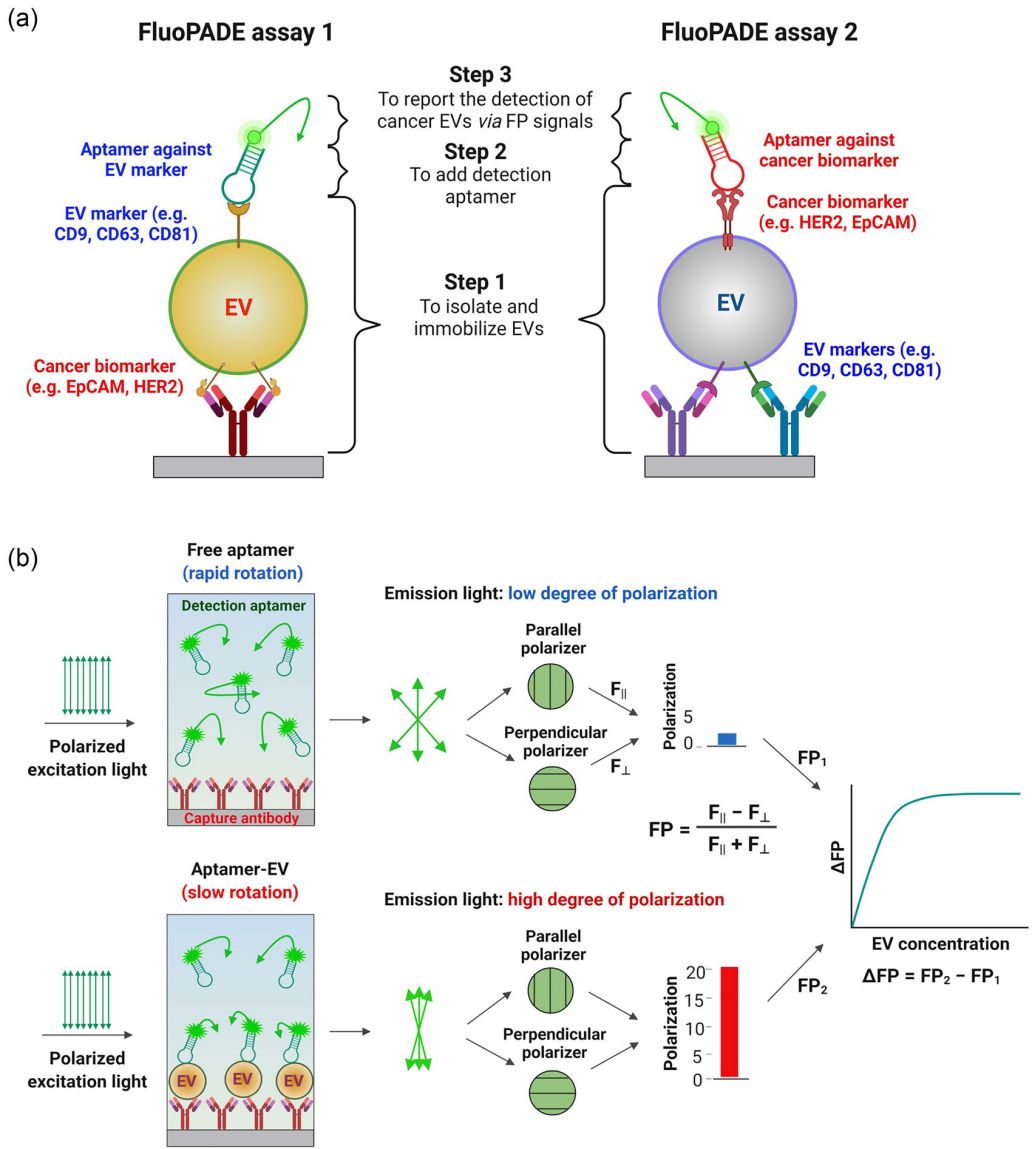
Illustration of FluoPADE assays. (a) The design of and the process involved in the FluoPADE assays. (b) Schematic illustration of working principle of fluorescence polarisation‐based cancer EV detection assay using fluorescently labelled aptamers (FluoPADE assay).

The novel system detects cancer‐derived EV via three steps: (1) immobilising EVs via EV surface proteins either by antibodies against general EV markers, that is, tetraspanins, or EV cancer biomarkers, followed by a quick wash to remove impurities, for example, free proteins, membrane fragments or lipoproteins; (2) the addition of fluorescein‐labelled DNA aptamer against either EV marker or cancer biomarker; (3) detecting and quantifying cancer‐derived EV via fluorescence polarisation in the microwells without washing (Scheme 1a). The employment of different markers for capturing and detecting ensures that the recorded FP signal is indeed originated from cancer cell line‐derived EVs but not from free proteins, membrane fragments or protein aggregates. Scheme 1b depicts the working principle of the FluoPADE assays.

### 3.2 Optimisation of conditions in FluoPADE assays

The aim of this study was to develop FluoPADE assays to selectively and sensitively detect EVs of cancer origin. Thus, two different sets of EV‐capturing antibodies were employed in the assays to immobilise EVs onto the solid support by using antibodies against either tetraspanins as markers for EVs or cancer biomarkers. Specifically, biotinylated anti‐human EpCAM antibody was used to capture EVs derived from carcinoma cells, for example, HT‐29 colorectal carcinoma cells. Alternatively, as a prototype of pan‐EV capture, a mixture of biotinylated antibodies against human CD9 and CD81 were used to immobilise EVs from SKBR3 breast carcinoma cells onto a black streptavidin‐coated 96‐well plate. As FluoPADE assays are based on the EV isolation by capturing antibodies in the microwell, thus, the bovine serum albumin and Tween 20 at very low concentration in Washing buffer‐A were used before and during EV capturing for blocking non‐specific EV capture in the microwell. It was then proceeded to confirm that the platform consisted of biotinylated antibodies and streptavidin‐coated microplate does enable the effective isolation of EVs from cell culture conditioned medium.

**FIGURE 1:**
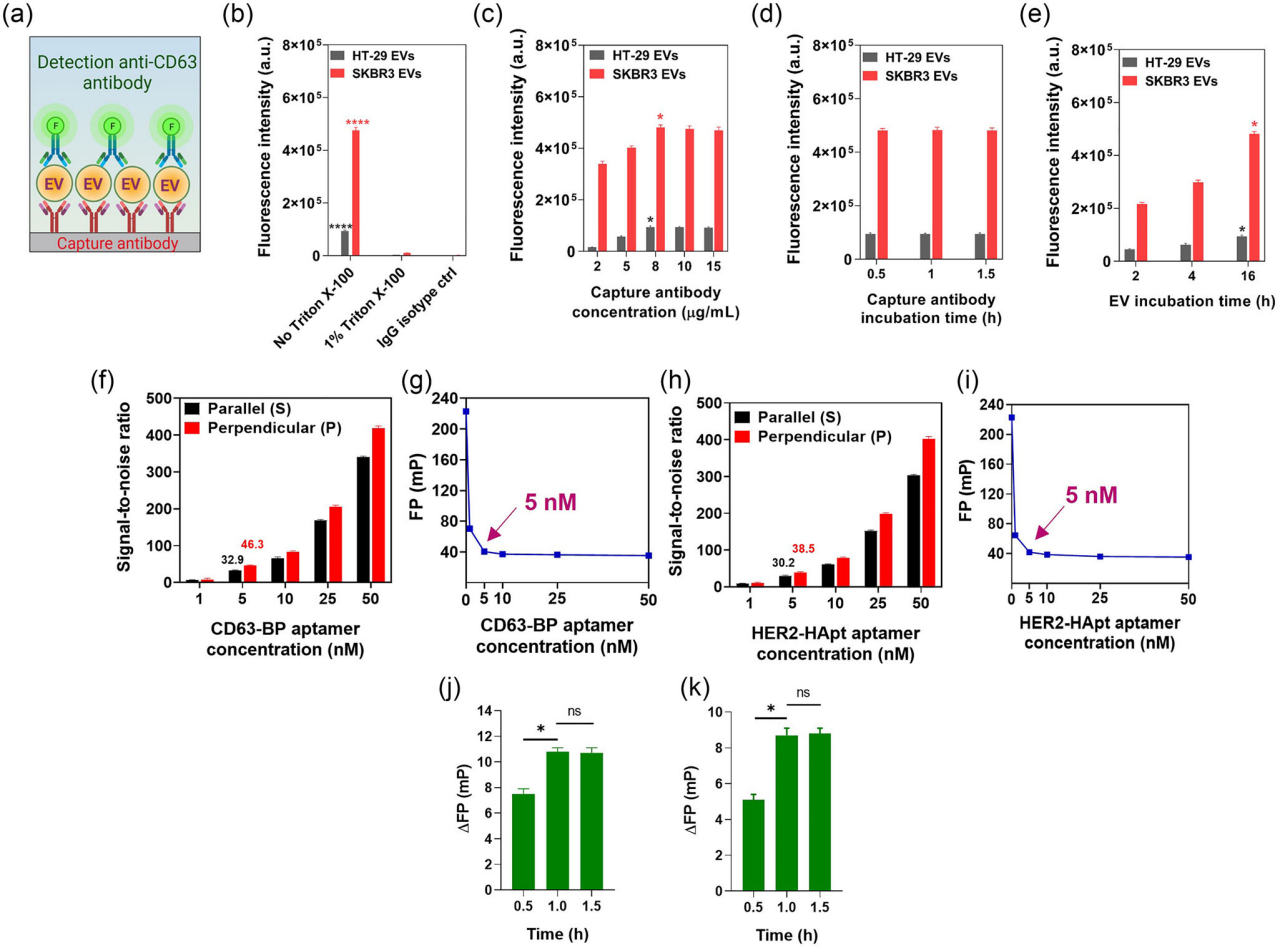
Optimisations of the FluoPADE assays. (a‒e), Optimisation of EV isolation. (a) Schematic illustration of EV immobilisation using antibodies followed by the detection using FITC‐conjugated anti‐CD63 antibody. (b) HT‐29 EVs were captured using biotinylated anti‐EpCAM antibody, EVs from SKRB3 cells were captured using 1:1 molar ratio of biotinylated anti‐CD9/CD81 antibodies. As a control of particles versus vesicles, the captured EVs were treated with 1% Triton X‐100 or saline prior to immobilisation. **** *p *< 0.0001 compared with EVs treated with Triton X‐100. (c) Optimisation of concentration of biotinylated anti‐EpCAM antibody for HT‐29 EV capture and that of biotinylated anti‐CD9/CD81 antibodies for capturing EVs from SKBR3 cells. * *p *< 0.05 compared with fluorescence intensity for EVs immobilised using 5 µg/mL antibody. (d) Optimisation of well‐coating time for biotinylated anti‐EpCAM antibody for HT‐29 EV capture and that for the mixture of anti‐CD9/CD81 antibodies for SKBR3 EV capture. (e) Optimisation of capture time for HT‐29 EVs by anti‐EpCAM antibody or SKBR3 EVs by anti‐CD9/CD81antibodies. * *p *< 0.05 compared with fluorescence intensity of the group of 4 h incubation. (f‒i) Optimisation of aptamer concentration. (f) Signal‐to‐noise ratios of parallel and perpendicular fluorescence intensity for free FITC‐labelled CD63‐BP aptamer at various concentrations over that of EV sample without aptamers. (g) FP of free FITC‐labelled CD63‐BP aptamer at the concentrations indicated in the absence of EVs. (h) Signal‐to‐noise ratios of parallel and perpendicular intensity for free FITC‐labelled HER2‐HApt aptamer at the concentrations indicated over that of EV sample without aptamers. (i) FP of FITC‐labelled HER2‐HApt aptamer at the concentrations indicated in the absence of EVs. (j and k) Optimisation of incubation time for aptamers and immobilised EV. (j) The fluorescence polarisation of FITC‐CD63‐BP aptamer added to the immobilised HT‐29 EVs. (k) The fluorescence polarisation of FITC‐HER2‐HApt aptamer added to the immobilised SKRB3 EVs. *p < 0.05; ns: not significant. Data shown are means ± S.D, *n* = 3.

As shown in Figure 1a, FITC‐labelled anti‐human CD63 antibody was used to detect immobilised EVs. Figure 1b shows the fluorescence intensity (FI) of anti‐human CD63 antibody for immobilised HT‐29 EVs and SKBR3 EVs with or without prior treatment with 1% Triton X‐100 at room temperature for 30 min so that EVs were lysed (>97%) then there were no intact EVs left after the treatment. For both HT‐29 EVs and SKBR3 EVs, the FI signals of CD63 for EVs immobilised without the prior treatment of Triton X‐100 (93,258.5 ± 3043.3 for HT‐29 EVs, 475,123.2 ± 11421.2 for SKBR3 EVs) were markedly higher (*p *< 0.0001) than that in samples treated with 1% Triton X‐100 (2736.2 ± 58.9 for HT‐29 EVs, 9694.6 ± 317.4 for SKBR3 EVs) or than that in the wells coated with an isotype‐matched anti‐IgG antibody (119.1 ± 15.7 for HT‐29 EVs, 1370.0 ± 143.7 for SKBR3 EVs) where no EV had been immobilised onto the plate (Figure 1b). These data firmly established the feasibility of efficient capture of EVs in our setup. Next, the optimal conditions for EV immobilisation were determined. First, the concentrations of capturing antibodies for EVs from HT‐29 using an antibody to EpCAM as well as that for capturing EVs from SKBR3 using anti‐CD9/CD81 antibodies at 1:1 ratio (weight/weight) were investigated in a range from 2.0 to 15.0 µg/mL. As shown in Figure 1c, in both cases, the signal for CD63 increased from the concentration of immobilisation antibody of 2.0–8.0 µg/mL. Beyond the concentration of 8.0 µg/mL, there was no discernible improvement in the signal for CD63. Hence, the optimal concentration for capturing EVs using biotinylated anti‐EpCAM antibody or biotinylated anti‐CD9/CD81 antibodies was determined as 8.0 µg/mL.

Next, the most optimal time needed for coating the capturing antibody onto the streptavidin‐coated microwell was determined. As shown in Figure 1d, there was no significant difference for signals among incubation time points tested when the capture antibodies were incubated with the streptavidin‐coated microwell for a length of 0.5, 1 or 1.5 h for either biotinylated EpCAM antibody or biotinylated CD9/CD81 antibodies, indicating a 0.5 h incubation is sufficient. Finally, the optimum time for incubation of EVs with immobilised capturing antibodies was found to be 16 h (Figure 1e).

Having optimised the conditions for immunoisolation of EVs, the optimal concentrations of FITC‐conjugated CD63‐BP aptamer and FITC‐conjugated HER2‐HApt aptamer in FluoPADE assays were demonstrated by their signal‐to‐noise (S/N) ratios for parallel and perpendicular fluorescence intensities and FP_aptamer_ values over a wide range of aptamer concentrations.

As shown in Figure 1f,h, the S/N ratios for parallel and perpendicular fluorescence intensity rose with the increasing concentrations of two aptamers. Whilst the ratios for parallel and perpendicular intensity increased from 6.9 ± 0.7 and 7.6 ± 3.7 at 1 nM to 341.2 ± 2.1 and 419.0 ± 5.9 at 50 nM for CD63‐BP aptamer, respectively (Figure 1f), the respective ratios for HER2‐HApt aptamer increased from 8.6 ± 0.8 and 10.6 ± 2.1 at 1 nM to 303.5 ± 2.3 and 402.3 ± 6.4 at 50 nM, respectively (Figure 1h). It has been well established that the optimal concentration of a ligand in a FP assay should be chosen at the lowest possible concentration at which the contributions of the parallel and perpendicular fluorescence intensities from the background to the final FP is insignificant so that the FP signal obtained is derived mainly from the detection ligand instead from the background noise (Moerke, 2009). In this study, the S/N ratios for both the parallel and perpendicular fluorescence intensities were higher than 30 at the concentration of 5 nM for the two aptamers studied (Figure 1f,h). With such high S/N ratios, the intensities for the parallel and perpendicular fluorescence derived from detection aptamers dominate the final FP signal. Moreover, the concentration of 5 nM was much lower than the *K_D_* of each aptamer (Figure S2), ensuring reasonable level of FP signals are detected (Jameson & Ross, 2010). Furthermore, FP values remained constant once the aptamer concentration reached 5 nM and beyond for both aptamers (Figure 1g,i). Therefore, the concentration of 5 nM for FITC‐labelled aptamers was chosen as the optimal concentration of detection ligand in our assays.

In order to achieve the best performance of FluoPADE assays, the incubation time of immobilised EVs with the detection aptamers was also optimised. As shown in Figure 1j, ΔFPs for CD63‐BP aptamer increased significantly from 7.5 ± 0.4 mP at 0.5 h time point to 10.8 ± 0.3 mP at 1.0 h (*p* < 0.05) with no further increase in ΔFP at 1.5 h (10.7 ± 0.4 mP). Similarly, the optimal incubation time for HER2‐HApt aptamer was found to be 1.0 h with the highest ΔFP of 8.7 ± 0.4 mP (Figure 1k). Therefore, the optimal time for incubating immobilised EVs with CD63‐BP aptamer and HER2‐HApt aptamer was determined as 1.0 h.

### 3.3 FluoPADE assays are specific

The selectivity and specificity are vital for a diagnostic assay, thus this work is aimed to establish highly specific FluoPADE assays. After the optimisation of the initial working conditions for setting up the assays, the selectivity and specificity of these detection methods on EpCAM‐positive EVs derived from HT‐29 cells and HER2‐postive EVs derived from SKBR3 cells were exhaustively characterised.

To demonstrate that the assays detect only EVs but not soluble membrane fragments or free proteins, a control in which immobilised EVs were treated with 1% Triton X‐100 for 30 min at room temperature for lysing intact EVs was included (Figure 1b). For the EV‐free and protein‐only control sample, 100 µL of 50 nM EpCAM protein was added to the wells to saturate the binding sites on capture EpCAM antibody (*K_D_* of 9.5 nM) to investigate the maximum non‐specific binding of CD63‐BP aptamer to the EpCAM protein. Further controls for specificity entailed control groups with immobilised EVs derived from cells that do not express EpCAM or HER2 to demonstrate that the FluoPADE assays detect EVs with specific biomarkers of interest. Another set of different controls employed included FITC‐conjugated negative control aptamer to rule out the potential interactions between fluorophore (FITC) molecules or the negative charge on nucleic acid and captured EVs and/or antibody‐coated well. Finally, an IgG isotype‐matched antibody‐coated well was used as a negative control to demonstrate that FP signals were derived from EVs but not from the assay matrix.

**FIGURE 2:**
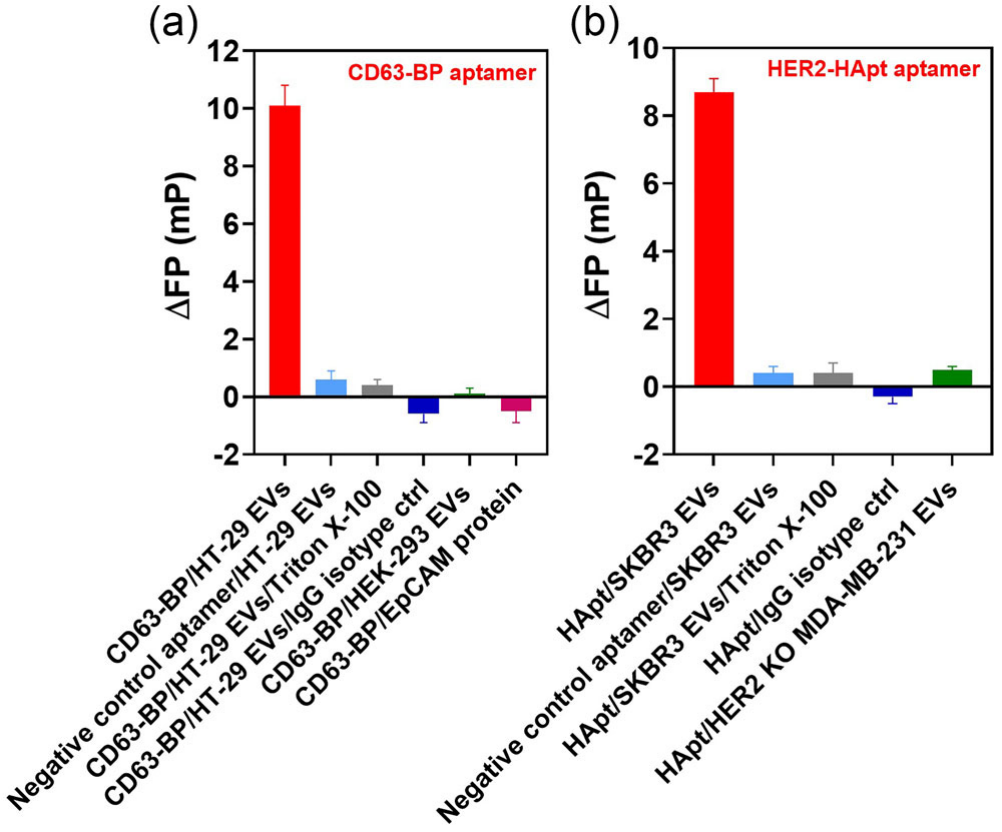
Specificity of FluoPADE assays. (a) Difference in fluorescence polarisation of 5 nM FITC‐CD63‐BP aptamer at a detection ligand against six different analytes: EVs from HT‐29 cells immobilised using anti‐EpCAM antibody; FITC‐negative control aptamer incubated with HT‐29 EVs immobilised using anti‐EpCAM antibody; HT‐29 EVs immobilised using anti‐EpCAM antibody followed by the lysis using 1% Triton X‐100; HT‐29 EVs incubated with the microwell coated with IgG isotype control antibody; EVs from HEK293 cells that do not express EpCAM; and immobilised EpCAM protein without the input EVs. (b) Difference in fluorescence polarisation of 5 nM FITC‐HER2‐HApt aptamer as a detection ligand against five different analytes: SKRB3 EVs immobilised using anti‐CD9/CD81 antibodies; FITC‐negative control aptamer incubated with SKRB3 EVs immobilised using anti‐CD9/CD81 antibodies; SKRB3 EVs immobilised using anti‐CD9/CD81 antibodies followed by the lysis with 1% Triton X‐100; SKRB3 EVs incubated with the microwell coated with isotype control antibody; HER2‐negative EVs from MDA‐MB‐231cells with the HER2 gene knocked out. Data shown as means ± S.D., *n* = 3.

In experiments shown in Figure 2a, most wells were coated with antibodies against EpCAM for the capture of EpCAM‐positive EVs from HT‐29 cells, except for the isotype‐matched negative control in which the anti‐IgG antibody was coated. The presence of captured EVs in our assay was clearly detected using FITC‐labelled CD63‐BP aptamer, evident from the ΔFP of 10.1 ± 0.7 mP for the wells containing immobilised EVs derived from HT‐29 cells. In contrast, minimal ΔFP (ΔFP ≤ 0.6 mP) was observed for the negative controls, including the negative control aptamer that does not bind to CD63, the sample treated with Triton X‐100 that lysed EVs, or EVs from HEK293 that does not express EpCAM (Figure 2a). In fact, the fluorescence polarisation changes for these controls also were lower than the limit of detection of this assay. These results were corroborated ΔFP of −0.6 ± 0.3 mP recorded for the sample with isotype‐matched antibody control, further demonstrating the high selectivity of the assay for EpCAM‐positive EVs. With the purpose of excluding the possibility of the false positive ΔFP resulted from the binding of CD63 aptamer to the free EpCAM protein immobilised onto anti‐EpCAM antibody coated well, a further negative control in which only the free EpCAM protein but no EpCAM‐positive EVs was investigated. As shown in Figure 2a, the ΔFP of −0.5 ± 0.4 mP for this negative control revealed that there was no interaction between the FITC‐CD63‐BP aptamer and the immobilised EpCAM protein in the microwells. Taken together, the results in Figure 2a clearly demonstrate that not only the assay is selective and specific to EpCAM‐positive HT29 EVs, but also there is a negligible contribution from the interactions between FITC, HT‐29 EVs and antibody‐coated well to the ΔFP recorded.

Next, the specificity of FP signals from FITC‐labelled HER2 DNA aptamer to EVs immobilised using anti‐CD9 and CD81 antibodies on the microwells was examined. In a similar pattern, a ΔFP of 8.7 ± 0.4 was detected for assays using HER2‐HApt aptamer from the intact HER2‐positive EVs, which was significantly higher than the ΔFPs from negative control samples including that from negative‐control aptamer, EVs that had been lysed by detergent, isotype‐matched control antibody or EVs from MDA‐MB‐231 cells with HER2 gene knocked out (*p* < 0.0001) (Figure 2b). Indeed, the ΔFPs of these negative controls were lower than the LOD of the assays.

In conclusion, FluoPADE assays using capture antibodies, for example, anti‐EpCAM antibody or a mixture of anti‐CD9/CD81 antibodies, and detection aptamers, for example, CD63‐BP aptamer or HER2‐HApt aptamer, are selective and specific to EpCAM‐positive EVs from HT‐29 cells and HER2‐positive EVs from SKBR3 cells EVs as originally designed.

### 3.4 The limit of detection and linear dynamic range of FluoPADE assays

Having optimised FluoPADE assays and demonstrated the specificity of the assays, the sensitivity and linear dynamic range of the assays were determined. To this end, the input EV in different matrices was prepared. For the initial characterisation, the EVs prepared from cancer cell lines were spiked into F‐PBS. For future application in liquid biopsy, the cancer cell line‐derived EVs were spiked into human plasma for the determination of the limit of detection and linear working range of the assay.

**FIGURE 3:**
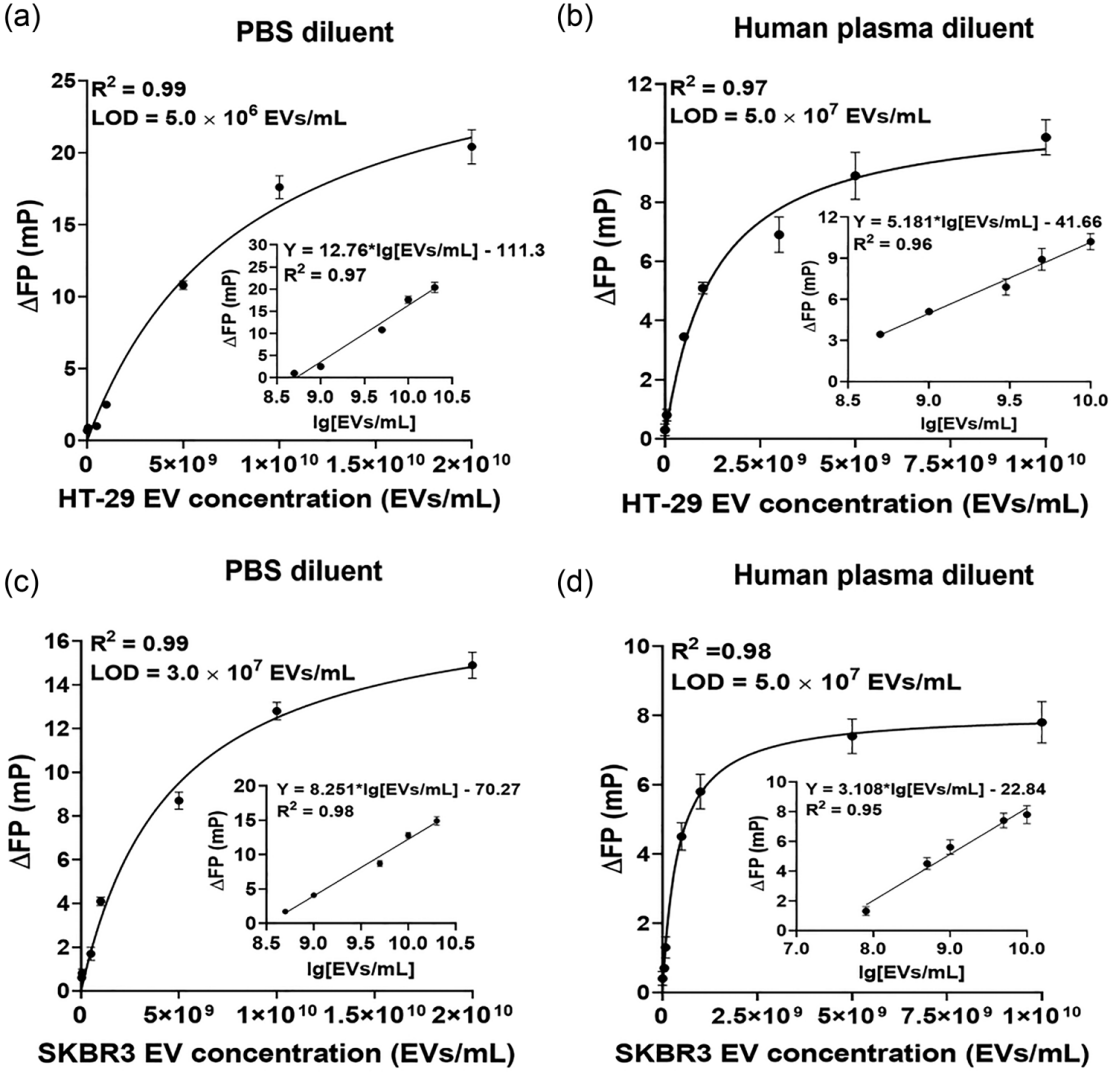
Limit of detection and linear dynamic range of FluoPADE assays. Changes in the fluorescence polarisation as a function of EV concentration and linear plots of fluorescence polarisation change as a function of the log10 (lg) of EV concentration (inset) are presented, along with limit of detection and linear dynamic range of FluoPADE assays. (a and b) CD63‐BP aptamer with EVs from HT‐29. (c and d) HER2‐HApt aptamer with EVs from SKBR3. Cell line‐derived EVs were diluted at a 1:9 ratio either in PBS (a, c) or in human plasma (b, d). Data shown are means ± S.D., *n* = 3.

In this work, the limit of detection (LOD) of FP assays was defined as the EV concentration at which the FP signal of the assay was equal to or higher than the sum of the respective blank FP signal plus three times the standard deviation of the blank signal ($LOD=\;Mean_{blank}+3\times \;SD_{blank})$. Figure 3 presents the LOD and linear dynamic range (LDR) of FluoPADE assay on EVs spiked into F‐PBS and into human plasma for each detection aptamer. Interestingly, FluoPADE assays using EVs spiked into human plasma exhibited higher LODs and downward LDRs as opposed to respective values and ranges of the assays on EVs diluted in phosphate‐buffered saline (comparing Figure 3a,c to Figure 3b,d, respectively). For CD63‐BP aptamer‐based FluoPADE assay, the LOD decreased from 5.0 × 10^6^ HT‐29 EVs/mL for EVs in saline to 5.0 × 10^7^ HT‐29 EVs/mL for EVs diluted in human plasma. In contrast, the LDR of 5.0 × 10^8^ ‒ 2.0 × 10^10^ HT‐29 EVs/mL for EVs spiked into saline was wider than that of 5.0 × 10^8^ to 1.0 × 10^10^ for EVs spiked into human plasma (Figure 3a,b). Similarly, in FluoPADE assay using HER2‐HApt aptamer, it had a slightly increased LOD of 5.0 × 10^7^ SKBR3 EVs/mL and a narrower LDR of 8.0 × 10^7^ ‒ 1.0 × 10^10^ SKBR3 EVs/mL for EVs spiked into human plasma as compared to the LOD of 3.0 × 10^7^ SKBR3 EVs/mL and LDR from 5.0 × 10^8^ to 2.0 × 10^10^ SKBR3 EVs/mL for the EVs in PBS (Figure 3c,d).

The differences in LODs and LDRs of the assays using identical detection aptamer between the same EVs suspended in saline and that into human plasma could be attributed to the differences in the total amount of marker proteins available in different samples. In sample with PBS as the assay matrix, the only EVs present were those derived from the cell line. In contrast, in the sample with plasma as the matrix, there the EVs consisted of a mixture of cell line‐derived EVs plus the native EVs in the human plasma (Figure S3). Therefore, the altered dynamic interactions between the fixed amount of immobilised EV capturing antibodies on the microwell and the different amounts of biomarker‐positive EVs in saline versus plasma would contribute to the disparate extent of fluorescence polarisation observed. Another factor could also contribute to the differences is that the movement and/or collision of other plasma‐derived EVs or particles may interfere with the binding of spiked‐in EVs with the capture antibody. Furthermore, protein corona on spiked‐in EVs could also play a role in affecting the capture of EVs since the corona layer formed on spiked‐in EV surface may mask the capture markers on the EVs, resulting in the reduction of the binding of the EVs to capture antibodies (Heidarzadeh et al., 2023; Tóth et al., 2021). Taken together, these factors could underlie the observed less efficient capture of spiked‐in EV in the presence of human plasma compared to that in PBS.

### 3.5 Fluorescence polarisation outperforms fluorescence intensity in detection and marker quantification of extracellular nanovesicles

Fluorescence intensity (FI)‐based assays have been widely utilised in fluorescence‐based quantitative assays in biochemistry and molecular cell biology. In contrast, fluorescence polarisation is seldom utilised as a mode of detection in cell biology in general and in EV studies in particular. Here, with the experimental set‐up of immobilised EVs, the performance of EV detection between fluorescence intensity‐based and fluorescence polarisation‐based assays using the identical detection aptamers or detection antibodies with LOD and linear dynamic range of detection as readouts were compared.

The relationships between FI and EV concentrations, LODs and LDRs are shown in Figure S4 for FI‐based assays using CD63‐BP aptamer and HER2‐HApt aptamer on EVs from HT29 or SKBR3 in F‐PBS (Figure S4a,c) or that spiked into human plasma (Figure S4b,d). The LOD of FI‐based assays for detecting cancer cell‐derived EVs in F‐PBS using FITC‐labelled CD63‐BP aptamer was determined as 2.0 × 10^8^ HT‐29 EVs/mL (Table 2), the LOD using FITC‐labelled HER2‐HApt aptamer was 5.0 × 10^8^ SKBR3 EVs/mL (Table 2). Not surprisingly, as compared to FI assays for EVs spiked in saline, the LOD of FI assays for EVs in human plasma was found to decrease by approximately 2.5‐fold to an LOD of 5.0 × 10^8^ HT29 EVs/mL for FI assay using CD63‐BP aptamer (Table 2), as well as to decrease by 2‐fold to an LOD of 1.0 × 10^9^ for FI‐based assay using HER2‐HApt aptamer (Table 2). The significant difference in LODs between cancer cell line‐derived EVs spiked in saline and that in human plasma could be partially attributed to the presence of additional amounts of biomarker proteins on EVs from human plasma as discussed in the preceding section. Interestingly, FluoPADE is superior in its sensitivity for the detection of EVs, evident from its much lower value of LODs compared to those in FI assays using the same detection aptamer. Specifically, using the CD63‐BP aptamer, the LODs of FluoPADE assay in PBS and human plasma were determined as 5.0 × 10^6^ HT‐29 EVs/mL and 5.0 × 10^7^ HT‐29 EVs/mL, respectively. In contrast, the LOD of FI assays for the same measurement was 2.0 × 10^8^ HT‐29 EVs/mL and 5.0 × 10^8^ HT‐29 EVs/mL, respectively. This translates to a 40‐fold and 10‐fold increase in sensitivity of FluoPADE assay over that of the FI assay with PBS or human plasma as the assay matrix, respectively. Similarly, FP‐based assays using HER2‐HApt aptamer detected SKBR3 EVs with an LODs of 3.0 × 10^7^ and 5.0 × 10^7^ SKBR3 EVs/mL in PBS or plasma, respectively, whilst the corresponding FI assays with the same HER2‐HApt aptamer had an LOD of 5.0 × 10^8^ and 1.0 × 10^9^ SKBR3 EVs/mL. Consistent with the study using CD63‐BP aptamer, these differences in LODs with HER2‐HApt aptamer denotes a 17‐fold or 26‐fold increased sensitivity FluoPADE assay than the corresponding FI assays with PBS or human plasma as the assay matrix, respectively.

**TABLE 2:** Summary of limits of detection (LOD), limit of quantification (LOQ) and linear dynamic ranges of FluoPADE assays and fluorescence intensity (FI) assays.

Method	Detection probe	EV source	LOD in PBS (EVs/mL)	LOQ in PBS (EVs/mL)	Linear range in PBS (EVs/mL)	LOD in plasma (EVs/mL)	LOQ in plasma (EVs/mL)	Linear range in plasma (EVs/mL)
FluoPADE	CD63‐BP aptamer	HT‐29	5.0 × 10^6^	1.7 × 10^7^	5.0 × 10^8^‒2.0 × 10^10^	5.0 × 10^7^	1.7 × 10^8^	5.0 × 10^8^‒1.0 × 10^10^
FluoPADE	HER2‐HApt aptamer	SKBR3	3.0 × 10^7^	1.0 × 10^8^	5.0 × 10^8^‒2.0 × 10^10^	5.0 × 10^7^	1.7 × 10^8^	8.0 × 10^7^‒1.0 × 10^10^
FI	CD63‐BP aptamer	HT‐29	2.0 × 10^8^	6.6 × 10^8^	3.0 × 10^8^‒2.0 × 10^9^	5.0 × 10^8^	1.7 × 10^9^	1.0 × 10^9^‒1.0 × 10^10^
FI	HER2‐HApt aptamer	SKBR3	5.0 × 10^8^	1.7 × 10^9^	1.0 × 10^9^‒2.0 × 10^10^	1.0 × 10^9^	3.3 × 10^9^	3.0 × 10^9^‒1.0 × 10^10^
FI	Anti‐CD63 antibody	HT‐29	1.0 × 10^7^	3.3 × 10^7^	5.0 × 10^7^‒1.0 × 10^9^	5.0 × 10^7^	1.7 × 10^8^	1.0 × 10^8^‒2.0 × 10^9^
FI	Anti‐HER2 antibody	SKBR3	1.0 × 10^9^	3.3 × 10^9^	2.0 × 10^9^‒2.0 × 10^10^	3.0 × 10^9^	1.0 × 10^10^	5.0 × 10^9^‒1.0 × 10^10^

*Note*: LOQ = 3.3 × LOD.

In addition to investigating the performance in detection of FI assays using aptamers, the performance of FI assays using FITC‐labelled CD63 antibody and FITC‐labelled HER2 antibody as the detection ligands was also studied despite the fact that these antibodies were unable to detect EVs by FP probably because their bulky sizes (Figure S2). Figure S5 presents the fitting of FI curves over EV concentration, LODs and LDRs in antibody‐based FI assays. For the detection of EVs in PBS, the LOD and LDR of fluorescence intensity assay employing anti‐CD63 antibody was 1.0 × 10^7^ HT‐29 EVs/mL and 5.0 × 10^7^ ‒ 1.0 × 10^9^ HT‐29 EVs/mL, respectively (Table 2), whilst the LOD and LDR using anti‐HER2 antibody was 1.0 × 10^9^ SKBR3 EVs/mL and 2.0 × 10^9^ ‒ 2.0 × 10^10^ SKBR3 EVs/mL, respectively (Table 2). For FI‐based assay detecting EVs in human plasma, the LOD and LDR employing anti‐CD63 antibody was 5.0 × 10^7^ HT‐29 EVs/mL and 1.0 × 10^8^ ‒ 2.0 × 10^9^ HT‐29 EVs/mL (Table 2), whilst the LOD and LDR with anti‐HER2 antibody was 3.0 × 10^9^ SKBR3 EVs/mL and 5.0 × 10^9^ ‒ 1.0 × 10^10^ SKBR3 EVs/mL, respectively (Table 2).

These results of LODs have firmly established that the FluoPADE assays using aptamers as detection ligands are superior to traditional fluorescence intensity‐based assays regardless of whether aptamers or antibodies are used to detect EVs based on the surface biomarkers in simple buffer as well as in human plasma. Furthermore, the FluoPADE assays are capable to quantify EV biomarkers in wider concentration ranges compared to FI‐based assays (Table 2).

### 3.6 FluoPADE assays are highly sensitive

One of the key challenges for EV‐based cancer diagnostics is the low abundance of tumour‐derived EVs in the clinical samples as the tumour‐derived EVs are released into a vast pool of EVs released by some 200 types of health human cells in biological fluids (Tian et al., 2018; Zhao et al., 2019). This equates to trying to find a needle in a haystack. To determine the sensitivity of CD63‐BP aptamer‐based FluoPADE assay, a serial titration was performed in which EpCAM‐positive EVs from HT‐29 cells were mixed with EpCAM‐negative EVs from HEK‐293 cells at five different ratios of 1:2000, 1:1000, 1:500, 1:100 and 1:10, with the total concentration of EVs maintained at 1.0005 × 10^10^ EVs/mL. For FluoPADE assay using HER2‐HApt aptamer, a serial titration of EVs from HER2‐positive SKBR3 cells and EVs from HER2 gene knockout MDA‐MB‐231 cells were prepared at ratios of 1:1000, 1:750, 1:500, 1:100 and 1:10, with the fixed total concentrations of EVs maintained at 3.0030 × 10^10^ EVs/mL. Of note, the total concentrations of EV were purposely maintained at a level of 10^10^ EVs/mL to mimic the physiological concentration of EVs in the systemic circulation (Johnsen et al., 2019), and thus the results from this study are clinically relevant and translatable.

**FIGURE 4:**
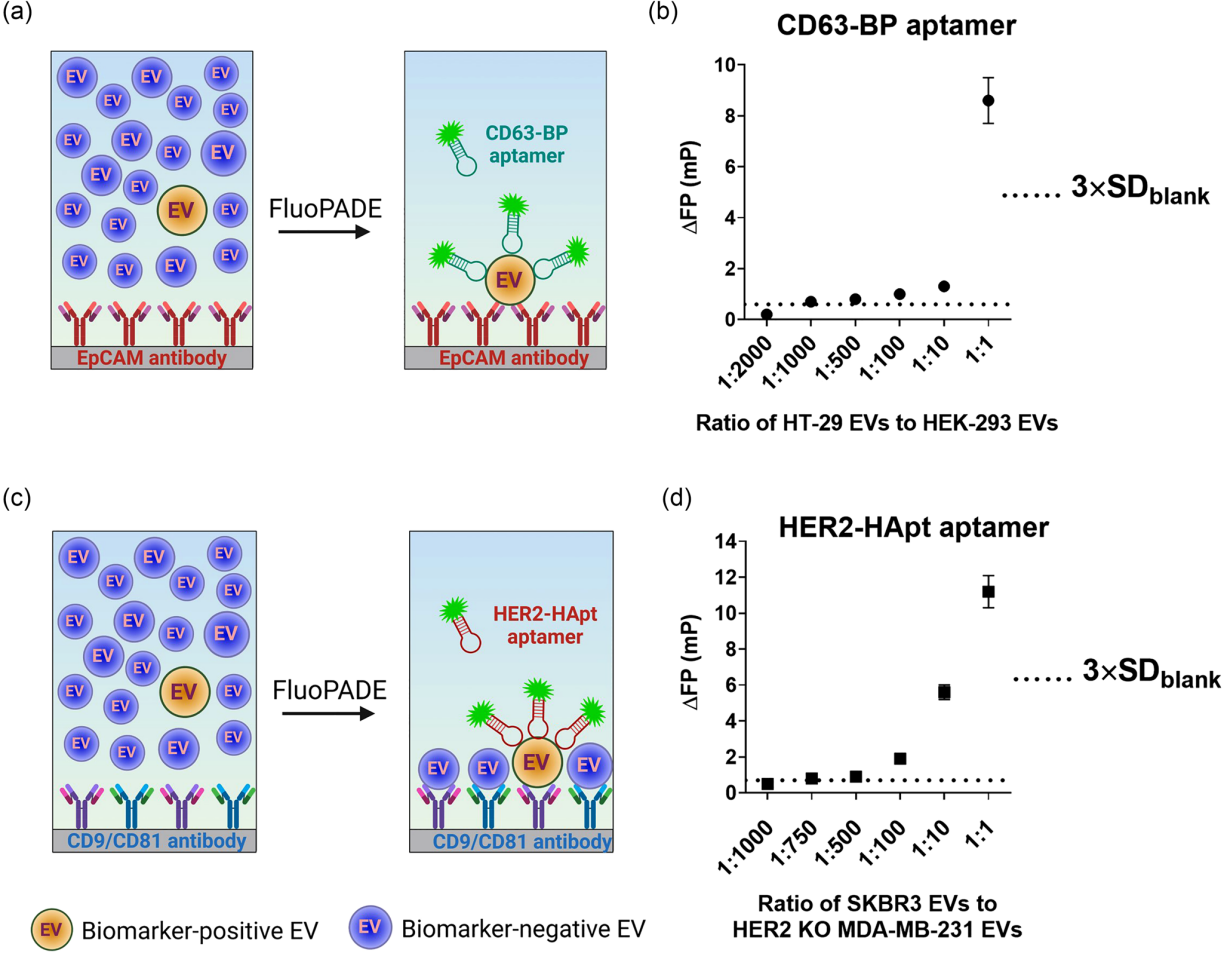
The sensitivity of FluoPADE assay in the detection of cancer biomarker‐positive EVs. Changes in fluorescence polarisation of aptamers as a function of decreasing prevalence of EVs that display a cancer biomarker protein on the EV membrane amidst a constant concentration of EV generated by serial dilution of the former with EVs that do not express the cancer biomarker protein. (a) Schematic illustration of the sensitivity assay using FITC‐CD63‐BP aptamer. (b) The ΔFP of serially diluted EpCAM‐positive HT‐29 EVs with EpCAM‐negative HEK293 EVs but maintaining the total EV concentration using FITC‐CD63‐BP aptamer. (c) Schematic illustration of the sensitivity assay using FITC‐HER2‐HApt aptamer. (d) The ΔFP of serially diluted HER2‐positive SKBR3 EVs with HER2‐negative Her2‐gene knockout MDA‐MB‐231 EVs but maintaining the total EV concentration using FITC‐HER2‐HApt aptamer. Data shown are means ± S.D., *n* = 3.

Practically, the sensitivity of our assay was defined as the lowest concentration ratio at which the observed ΔFP is ≥ 3 × SD_blank_. As shown in Figure 4, the ΔFP increased as the ratio of marker‐positive EVs to marker‐negative EVs increased whilst the total EV concentration remained constant. To detect EVs using CD63‐BP aptamer, ΔFP at ratio of 1:2000 was found to be lower than ΔFP of 3 × SD_blank_, that is, 0.2 ± 0.1 mP versus 0.6 ± 0.1 mP. However, ΔFP pattern of this assay witnessed a significant surge from 0.7 ± 0.1 at the EV ratio of 1:1000 to 8.6 ± 0.9 mP at the EV ratio of 1:1 (Figure 4b). This result indicates that the FluoPADE assay employing CD63‐BP aptamer is able to detect 1.0 × 10^7^ EpCAM‐positive HT29 EVs in the presence of 1000‐fold access of EpCAM‐negative EVs (1.0 × 10^10^ EpCAM‐negative EVs). The sensitivity of the HER2‐HApt aptamer‐based FluoPADE assay was established at a ratio of 1:750 of HER2‐positive EVs from SKBR3 cells to HER2‐negative EVs from Her2‐gene knockout MDA‐MB‐231 cells (Figure 4d).

### 3.7 The FluoPADE is capable of differentiating EVs from disparate sources via multiparametric analysis

A robust EV liquid biopsy assay should not only be highly sensitive but also capable of differentiating EVs from disparate sources using the same set of biomarkers. EVs released by the tumour at the primary site and metastatic sites are different entities both genetically and phenotypically. EVs released by cancer cells in a patient may all express a biomarker of interest qualitatively, however, it is conceivable that EVs released by the cancer cells at the different anatomical sites, for example, primary versus metastasis, at the different stages of tumour progression or in response to a given anticancer treatment will have quantitative differences in the abundance of the surface biomarker proteins. From diagnostic point of view, it will be highly beneficial that our FluoPADE is able to differentiate populations of EVs derived from different cancer cells based on the quantitative differences in their abundance of the surface biomarkers. In order to demonstrate the capacity of the FluoPADE to detect and/or differentiate EVs from difference origins, a model system in which EVs from three cell lines derived from three common types of solid cancers were used. The study began by determination of the differences in the abundance of CD63 and HER2 in the EVs prepared from colorectal cancer cells (HT‐29), breast adenocarcinoma cells (SKRB3) and hepatocellular carcinoma cells (HepG2) (Figure S6). It was hypothesised that the difference in the abundance of EV surface marker proteins can lead to the differences in the extent of fluorescence polarisation generated when our FITC‐labelled aptamer interacts with the EVs immobilised on a solid support. Therefore, the quantitative difference in the abundance of biomarkers on EVs from different cancer cell lines could be utilised for further classification of EVs from disparate origins via multiparametric analysis of FluoPADE assays.

**FIGURE 5:**
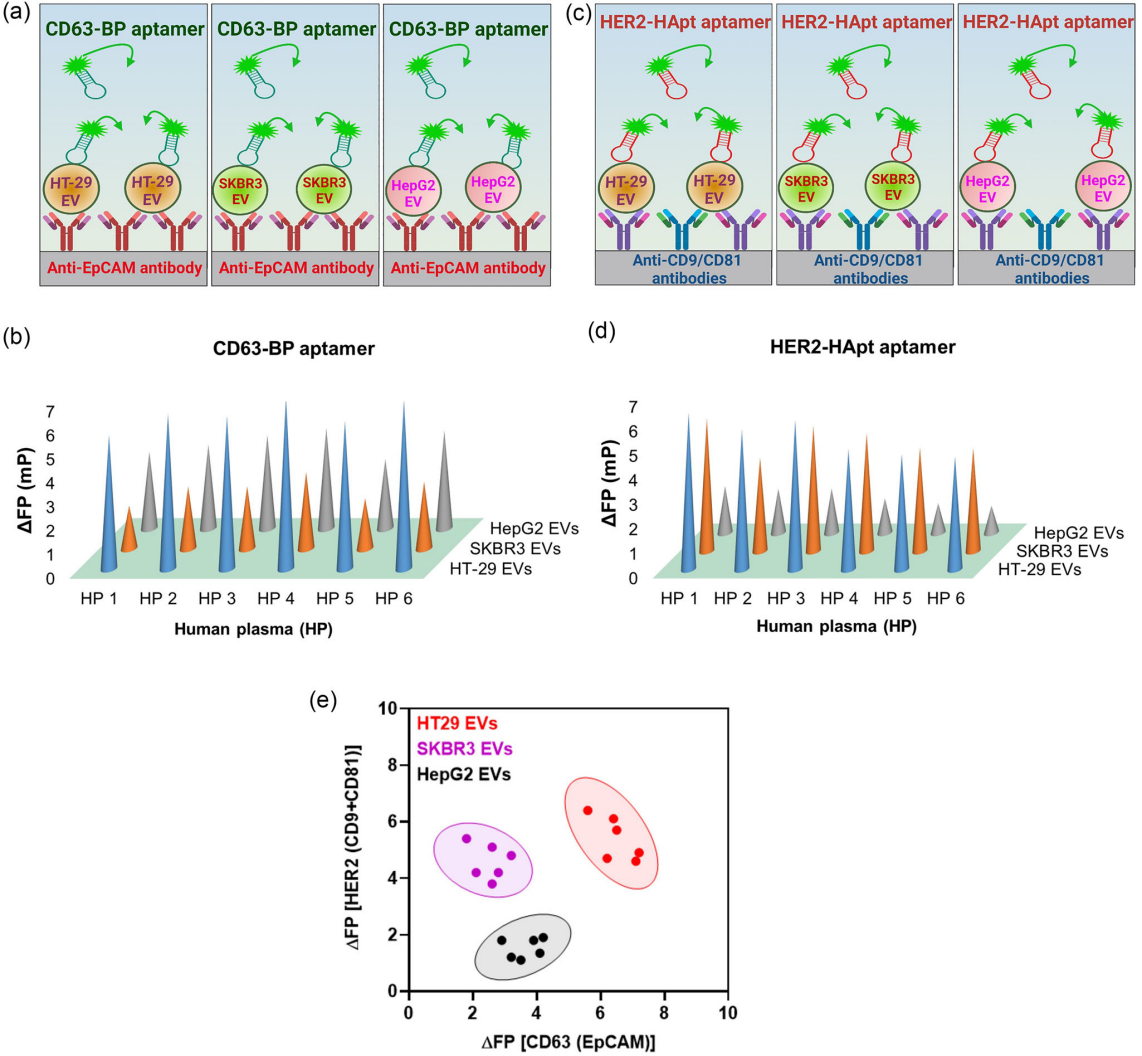
The FluoPADE is capable of differentiating EVs displaying the same set of biomarkers but from different sources via multiparametric analysis. (a) Schematic illustrations of FluoPADE assays detecting EVs immobilised using anti‐EpCAM antibody from three different cancer cell lines spiked into human plasma from six individual donors using FITC‐CD63‐BP aptamer. (b) Differential changes in fluorescence polarisation generated by the CD63‐BP aptamer on EVs immobilised with the same antibody to EpCAM from three different cancer cell lines spiked into human plasma from six individual donors. (c) Schematic illustrations of FluoPADE assays detecting EVs immobilised using anti‐CD9/CD81 antibodies from three different cancer cell lines spiked into human plasma from six individual donors with HER2‐HApt aptamer. (d) Differential changes in fluorescence polarisation generated by the HER2‐HApt aptamer on EVs immobilised with the same set of antibodies to CD9/CD81 from three different cancer cell lines spiked into human plasma from six individual donors. (e), Clustering plot of HT‐29 EVs, SKBR3 EVs and HepG2 EVs prepared from ΔFPs using CD63 aptamer as in (b) and ΔFPs using HER2 aptamer as in (d). Data shown are representative of three independent experiments. In all samples, the total EV concentration was maintained at 1.0 × 10^10^ EVs/mL; whilst spiked cancer cell line‐derived EVs were kept at 1.0 × 10^9^ EVs/mL.

To ensure the FluoPADE results on EVs prepared from cancer cell lines are not only relevant to the diagnostic setting of liquid biopsy, but also highly robust against possible interference from various elements in the blood plasma in individual patient, the analytes were carefully prepared by spiking the same number of EVs prepared from cancer cell lines with human plasma from six different blood donors. To this end, the cancer cell line‐derived EVs (1.0 × 10^8^ EVs) prepared from human colorectal cancer cells (HT‐29), breast cancer cells (SKRB3) and hepatocellular carcinoma cells (HepG2) were spiked into 9.0 × 10^8^ EVs of each of the six different human plasma samples in each microwell, resulting in a final EV concentration of 1.0 × 10^10^ EVs/mL for all 18 samples for each detection aptamer. The FITC‐labelled aptamer against EV marker (CD63) or FITC‐labelled aptamer against cancer biomarker (HER2) was utilised to detect EVs from three different types of solid cancers, each spiked into the plasma from six individual subjects as illustrated in Figure 5a,c. Interestingly, despite the inter‐plasma variations amongst EVs of the same cellular origin, the change of fluorescence polarisation for the detection aptamer against CD63 can demarcate the EVs immobilised with anti‐EpCAM antibody from three different cancer cells (Figure 5b). Specifically, HT‐29 EVs exhibited the highest ΔFP, followed by HepG2 EVs and SKBR3 EVs. These results were in line with the levels of CD63 expressed on HT‐29 EVs, HepG2 EVs and SKBR3 EVs immobilised onto anti‐EpCAM antibody‐coated magnetic beads (Figure S6). To test the reliability and robustness of the assay, a set of reciprocal experiments were carried out, in which the EVs from three different types of cancer were immobilised using antibodies against two EV markers, that is, CD9 and CD81, whilst the aptamer against HER2 was used as the detection probe for cancer biomarker. As shown in Figure 5d, ΔFPs for HT‐29 EV‐spiked human plasmas were higher than the values for SKBR3 EVs‐spiked human plasmas, whereas ΔFPs from HepG2 EV‐spiked human plasmas were the lowest among the EVs from three different sources. Again, these data were consistent with the expression levels of HER2 protein on HT‐29 EVs, SKBR3 EVs and HepG2 EVs immobilised onto magnetic beads coated with anti‐CD9/CD81 antibodies (Figure S6). Most importantly, when the ΔFP for individual analyte in these two sets of reciprocal measurements were plotted in a 2‐dimensional chart (Figure 5e), the distinct patterns of three populations of cancer‐derived EVs from three different cellular origins became apparent. These results underscored the highly attractive diagnostic utilities of the FluoPADE as it can not only detect cancer‐derived EVs with high sensitivity but also discern different populations of EVs characterised by the different abundances of the same biomarkers on the surface of EVs.

## 4 DISCUSSION

With the emerging potential of EVs as a new source of biomarkers for cancer liquid biopsy, remarkable efforts have been made to develop assays for detection and quantification of EVs of cancer origin (Min et al., 2021). Many analytical methods reported in the literature are sensitive, however, their inherent limitations hinder their translation into EV research laboratories and the clinic. Such limitations include, but are not limited to, the requisite intensive labour, the requirement of demanding biomedical engineering, sophisticated and expensive instruments needed to read and analyse signals, and high background from biological samples such as blood plasma. With a simple three‐step strategy, high specificity of capture antibodies and detection aptamers, FluoPADE assays are expected to replace ELISA assays to measure EV concentration with high sensitivity.

In the case of establishing a novel sensitive EV detection method, we used NTA to enumerate EV concentration in various samples. As NTA physically detects particles not vesicles, we compared the NTA counts in the samples before and after subjecting the samples to lysis with detergent. Thus, a vesicle concentration can be estimated. To this end, it is in line with many other EV research groups for the purpose of lysing EVs (Botha et al., 2022; Liu et al., 2022; Tian et al., 2019), 1% of Triton X‐100 was found to be optimal to lyse cell culture‐derived EVs (>97%, data not shown), thus EV lysed with 1% Triton X‐100 was employed as negative controls in our FluoPADE assays. However, Triton X‐100 at 0.5% (∼94% EVs lysed, data not shown) rather than 1% was selected for cell culture‐derived EV counting via NTA as the assay will not be conducted successfully when Triton X‐100 is used at higher 0.5% due to very high scattering light which will interfere NTA measurements. Caution must be exercised when using detergent in plasma‐derived EVs because of the presence of overwhelming number of lipoprotein particles in the blood (Botha et al., 2022; Tian et al., 2019). It has been demonstrated that detergents including Triton X‐100 (Botha et al., 2022; Sódar et al., 2016) can lyse or partially lyse lipoproteins and thus alter the total number of lipoprotein particles, resulting in aberrant NTA profile and total particle counts (Ikai & Hasegawa, 1978; Nielsen et al., 2006; Pownall, 2006). For example, Sódar et al. (2016) demonstrated that low‐density lipoproteins (LDL) are partially sensitive to Triton X‐100 at 0.1% (reduction of ∼15% of LDL counts), whereas Osteikoetxea and colleagues showed that Triton X‐100 at 0.075% could lyse from ∼88% to 96% EVs derived from four different cell lines including Jurkat, THP‐1, U937 and MiaPaCa (Osteikoetxea et al., 2015). To strike a balance between the lysis of EVs and breakdown of lipoproteins, we elected to use Triton X‐100 at 0.075% for the lysing of vesicles for plasma samples in NTA measurements.

One of the key challenges facing liquid biopsy of cancer‐derived EV is the co‐existence of cancer‐derived EVs amongst EVs from many different types of normal cells as well as other non‐EV components (e.g., soluble proteins, protein aggregates, non‐vesicular particles, membrane debris) in bodily fluids, cell culture media and even isolated EVs. Therefore, the specificity of an assay towards cancer biomarker‐positive EVs is a crucial factor that dictates its biomedical and clinical utility. Our FluoPADE assays were initially developed with a heavy emphasis on high specificity to EpCAM‐ and HER2‐derived EVs. Several strategies were employed to ensure the specificity of FluoPADE assays towards cancer biomarker‐positive EVs. First, the specificities of antibodies used to immobilise EVs, that is, anti‐EpCAM antibody or a mixture of anti‐CD9/CD81 antibodies followed by extensive washing laid the solid foundation of the specificity. Second, well‐characterised aptamers against either EV markers or cancer biomarkers were employed as detection ligands. This two‐pronged strategy would have removed most, if not all, impurities in the original input samples of biofluids, including soluble proteins, protein aggregates or non‐vesicular particles, by isolating and immobilising EVs before the detection step (Scheme 1a). In short, the specificity for cancer biomarkers is achieved via antibodies used to isolate EVs followed by the addition of detection aptamers. The removal of non‐EV components, coupled with the utilisation of a distinct set of ligands against biomarkers in EV capturing and detection, ensures that the observed signals originate mostly or exclusively from the captured EVs (Figure 2). In this context, there are currently only two published studies using FP to detect EVs. The first was reported by Kalimuthu and colleagues who employed 5‐dodecanoylamino fluorescein (C12‐FAM, a lipophilic dye) as a probe to detect EVs derived from HT‐29 cell line and TCMK1 cell line. However, the aggregation of dye molecules as well as the non‐specific interactions between C12‐FAM and non‐EV partners such as membrane debris or lipoproteins in the EV preparation may impede the specificity of the observed FP signals (Kalimuthu et al., 2019). In the second report, a CD63 aptamer was used to directly detect EVs from the A549 cell line prepared by ultracentrifugation or EVs in diluted human plasma (100X) without prior EV isolation (Zhang et al., 2019). Though the authors in this study demonstrated that their assay was much more sensitive to the CD63‐positive EVs than the soluble CD63 protein, the interaction between the probe and the soluble proteins and/or protein aggregates in EV preparation or in the plasma might limit its utility in cancer diagnostics. This is due to the extremely low limit of detection of aptamer‐based FP assays, such as LODs of 86 aM or 28 aM (Zhao et al., 2020), at which soluble proteins and protein aggregates in EV preparation or human blood can be readily detected as well.

Regarding the LOD and linear range of FluoPADE assays working with human plasma as the assay matrix, the assay could detect EpCAM‐positive EVs from HT‐29 cells at a concentration of 5.0 × 10^7^ EVs/mL with a linear range of quantification being 5.0 × 10^8^ to 1.0 × 10^10^ HT‐29 EVs/mL. For FluoPADE assays detecting HER2‐positive EVs from SKBR3 cells using HER2‐HApt aptamer, the LOD and linear range is 5.0 × 10^7^ SKBR3 EVs/mL and 8.0 × 10^7^‒1.0 × 10^10^ SKBR3 EVs/mL, respectively (Figure 3). Of note, some published assays using more complex devices and often more expensive instruments have reported LODs lower than those of FluoPADE assays as described above (Table 3). The FluoPADE assays can be used to detect and quantify cancer‐derived EVs in clinical samples, that is, human plasma. From a clinical perspective, the concentration of biomarker‐positive EV, for example, vascular endothelial cadherin‐positive EVs, in 25 patients bearing colorectal cancer are in a range from 5.0 × 10^8^/mL to 9.0 × 10^8^/mL (Bar‐Sela et al., 2020); whilst EV concentrations of healthy subjects or patients with HER2^+^ breast cancer are around 3 × 10^8^/mL and 10 × 10^8^/mL, respectively (Liu et al., 2019). As demonstrated in Figure 3 and Table 2, hence, our FluoPADE assays are generally much more sensitive and could quantify EVs in wider linear ranges as compared to many current ELISA‐based EV assays reported in the literature as well as those commercially available kits (Table 3).

**TABLE 3:** Summary of features of EV detection assays.

Method	Marker	LOD (EVs/mL)	Linear range (EVs/mL)	Probe washing	Signal stability	Reference
Dark field microscope	CD63, CD81	1.0 × 10^3^	1.0 × 10^3^‒1.0 × 10^7^	Yes	–	Yang et al. (2020)
Luminescence resonance energy transfer	CD63	1.1 × 10^6^	1.0 × 10^7^‒1.0 × 10^11^	Yes	‐	Chen et al. (2018)
Fluorescence	CD63	4.0 × 10^8^	4.0 × 10^8^‒4.0 × 10^10^	No	Real‐time	Ren et al. (2022)
Colorimetry	CD63	1.4 × 10^9^	1.9 × 10^9^‒3.4 × 10^10^	No	–	Wang et al. (2017)
Surface plasmon resonance	EGFR	2.0 × 10^10^	–	–	–	Liu et al. (2018)
Surface plasmon resonance imaging	HER2	2.0 × 10^8^	2.1 × 10^8^‒1.7 × 10^9^	No	Real‐time	Gool et al. (2017)
ELISA	CD81	2.0 × 10^9^	3.2 × 10^9^‒5 × 10^10^	Yes	<15 min	Zarovni et al. (2015)
ELISA	EGFR	1.0 × 10^10^	–	Yes	<15 min	Liu et al. (2018)
ELISA	CD81	4.1 × 10^9^	4.1 × 10^9^‒2.6 × 10^11^	Yes	<15 min	Kilic et al. (2018), ExoELISA™‐ULTRA CD81
ELISA	CD63	1.4 × 10^9^	1.4 × 10^9^‒4.6 × 10^10^	Yes	<15 min	ExoELISA™‐ULTRA CD63
ELISA	General EVs	2.0 × 10^8^	2.0 × 10^8^‒1.3 × 10^10^	No	<15 min	EXOCET™
Fluorescence polarisation	EpCAM‐CD63	5.0 × 10^6^	5.0 × 10^8^‒2.0 × 10^10^	No	Stable for at least 2 h (Table S1)	This study
Fluorescence polarisation	CD9/CD81‐HER2	3.0 × 10^7^	5.0 × 10^8^‒2.0 × 10^10^	No	Stable for at least 2 h (Table S1)	This study

The challenge brought by the dilution of cancer‐derived EVs by vast number of EVs released from normal cells is accompanied by another impediment in chemical pathology, which is the ability of a method to identify true positives and capture even subtle or low‐level changes. To achieve desired sensitivity, a number of sophisticated methods have been developed with a variety of detection strategies to meet this challenge by targeting proteins, for example, EpCAM, HER2, CD63, PSA and PTK7 (Liu et al., 2019; Yildizhan et al., 2021), and/or nucleic acids, for example, miRNA‐21, miRNA‐155, miRNA‐205 and miRNA let‐7b (Mahmudunnabi et al., 2022). For instance, Liu and colleagues employed thermophoretic enrichment and linear discriminant analysis together with a panel of seven aptamers to profile EVs collected from serum of patients with six different cancer types including breast, liver, lung, lymph, ovary and prostate cancers (Liu et al., 2019). The assay was able to detect Stage I cancer with 95% sensitivity and 100% specificity (Liu et al., 2019). Moreover, the different types of cancer could be differentiated with an overall accuracy of 68% (Liu et al., 2019). Other groups have reported that the concentration of EpCAM‐positive EVs collected from breast cancer patients increased from 6.36 × 10^8^ EVs/µL at Stage I‐II to 8.95 × 10^8^ EVs/µL at Stage III–IV (Kashefi‐Kheyrabadi et al., 2020). In this report, the aim was to develop separate FluoPADE assays based on two different working strategies to detect colorectal cancer‐derived EVs (HT‐29 EVs) and breast cancer‐derived EVs (SKBR3 EVs). Through careful optimisation of several key technical factors including EV immobilisation, detection probes, the optimal concentration of detecting aptamers as well as the length of incubation for the detecting aptamer and the immobilised EVs (Figure 1), FluoPADE assays exhibit high sensitivities. As such, our FluoPADE assays are able to recognise one HT‐29 EVs amidst 1000 EpCAM‐negative EVs from HEK‐293 cells using CD63‐BP aptamer, and one SKBR3 EVs in the background of 750 HER2‐negative EVs from HER2 gene knockout MDA‐MB‐231 cells using HER2‐HApt aptamer (Figure 4). Interestingly, Yildizhan et al. challenged their fibre optic surface plasmon resonance‐based assays to detect HEK‐293 EVs in 10% exosome‐depleted foetal bovine serum (EDS) as well as MCF7‐derived EVs in 100X‐diluted blood plasma. Although the authors did not provide the EV concentration of the matrices, however based on the data in the literature for EDS (Pham et al., 2021) and human plasma (Leggio et al., 2023), the estimated ratios of added‐in EVs to the background EVs would be around 1:0.01 to 1:0.1 (Yildizhan et al., 2021). Yang et al. developed a high throughput droplet digital ELISA (DEVA) for ultrasensitive single EV detection (Yang et al., 2022). In their endeavour to detect human neuron EVs spiked into foetal bovine serum, their DEVA could detect 11 human CD81‐positve EVs in the presence of 2 × 10^5^ bovine EVs, resulting in a ratio of 1 CD81‐positive EVs to 1.8 × 10^4^ CD81‐negative EVs (Yang et al., 2022). Of note, FluoPADE assay cannot detect EpCAM‐positive EVs from HT‐29 in the pool of EpCAM‐negative HEK‐293 EVs at the ratio of 1:2000 (Figure 4a). This is probably due to reduced random diffusion of EVs at this ratio. Specifically, the higher concentration of HEK‐293 EVs would likely decrease the degree of Brownian movement of HT‐29 EVs, resulting in the reduction of productive collision between a very low number of HT‐29 EVs and the capture EpCAM antibodies during EV capture. This dynamic movement of diluent EVs from plasma could be one of reasons behind the higher LODs of FluoPADE assays on EVs spiked in human plasma as compared to that in PBS (Figure 3, Table 2). This increase in LOD when EVs are spiked in complex matrix such as foetal bovine serum has also been demonstrated by Yang et al. (2022). The interaction between pre‐existing markers on plasma‐derived EVs (Figure S3) or markers on spiked‐in EVs and capture antibodies and/or plasma protein corona on spiked‐in EVs will result in the requirement of higher amounts of target EVs in the input analyte in order to achieve reliable detection. Pre‐existing EV biomarker proteins in human plasma have been demonstrated in the literature, such as CD63 (Logozzi et al., 2009; Salmond et al., 2021), CD9 (Salmond et al., 2021), CD81 (Rikkert et al., 2020), EpCAM (Amrollahi et al., 2019) and HER2 (Rikkert et al., 2020). These markers are found as transmembrane proteins on EVs from blood cells, epithelial cells or/and endothelial cells.

Although fluorescence intensity has been commonly used in fluorescence‐based quantitative analyses, this report has demonstrated that fluorescence polarisation outstripped fluorescence intensity in detecting and quantifying cancer EVs with the same experimental setup (Table 2). Furthermore, whilst antibodies were unable to detect cancer EVs through FP (Figure S2), these bulky probes also exhibited inferior detecting and quantifying performance of cancer EVs via fluorescence intensity in comparison with the performance of FluoPADE assays (Table 2). These findings suggest the superior ability of fluorescence polarisation using detection aptamers over fluorescence intensity using aptamers as detection probes in detecting and quantifying EVs. Moreover, fluorescence intensity assay can be affected by inner filter effects, autofluorescence, fluorescence quenching and photobleaching in samples at much greater degrees than FP assay (Corporation, 2006; Zhang et al., 2019), possibly resulting in lower reproducibility and precision for FI‐based assays. Collectively, along with the mentioned advantages from the homogeneity of the fluorescence polarisation method, this technique can be the next‐generation fluorescence‐based measurement quantity in EV‐based liquid biopsy both for EV research and clinical use in the future.

The biochemical and physical properties of cancer‐derived EVs, such as concentration, the qualitative and quantitative differences in biomarkers on EV, size and shape do vary amongst EVs from different types of cancer (Figure S1), different stages of cancer progression and response to treatment in individual patients (Melo et al., 2015; Willms et al., 2018). Therefore, multiparametric analyses on these variables are imperative for deciphering the heterogeneity of EVs from cancer patients (Liu et al., 2019). The FluoPADE assays presented here may or may not differentiate between one type of cancer that produces high amounts of EVs with low abundance of a given cancer biomarker from another type of cancer that produce less EVs with a higher surface density of the same cancer. However, in addition to the low detection limit and wider dynamic range, the FluoPADE assay is poised to be a valuable tool for real‐time personalised medicine aid to oncologists. As demonstrated in Figure 5, the multiple parametric format of the FluoPADE could be utilised to monitor the emergence of new macro‐ or micro‐metastasis, the clinical progression of cancer as well as the response to treatment through the quantification of relatively small changes in the concentration of EV‐based biomarkers during the entire clinical stages of cancer management. Recently, azadioxatriangulenium (ADOTA) dyes with a fluorescence lifetime of ∼20 ns and enhanced photostability have been reported (Panagopoulou et al., 2020). Compared with the shorter fluorescence lifetime (∼4 ns) of FITC, the labelling of aptamers with these new ADOTA dyes could significantly amplify FP signals in FluoPADE assays. Thus, with further improvement, the higher sensitivity of the FluoPADE assays in conjunction with their multi‐parametric analysis capabilities can be exploited for early cancer detection, for the detection of micro‐metastasis or for monitoring minimal residual disease.

## AUTHOR CONTRIBUTIONS

Cuong Viet Pham: Conceptualization (equal); data curation (equal); formal analysis (lead); investigation (lead); methodology (lead); software (equal); validation (lead); visualization (lead); writing—original draft (lead); writing—review and editing (lead). Rocky Chowdhury: Data curation (equal); formal analysis (equal); methodology (equal). Shweta Patel: Data curation (equal); formal analysis (equal); methodology (equal). Satendra Kumar Jaysawal: Data curation (equal); formal analysis (equal); methodology (equal). Yingchu Hou: Formal analysis (equal); writing—original draft (equal). Huo Xu: Visualization (equal); writing—review and editing (equal). Lee Jia: Visualization (equal); writing—review and editing (equal). Yu‐mei Zhang: Data curation (equal); formal analysis (equal); methodology (equal). Xiaowei Wang: Data curation (equal); resources (equal); visualization (equal). Wei Duan: Conceptualization (lead); data curation (equal); formal analysis (equal); funding acquisition (lead); methodology (equal); project administration (equal); resources (lead); supervision (lead); visualization (equal); writing—original draft (lead); writing—review and editing (lead). Dongxi Xiang: Conceptualization (lead); data curation (equal); formal analysis (equal); funding acquisition (equal); methodology (lead); project administration (equal); resources (equal); supervision (equal); validation (equal); visualization (equal); writing—original draft (lead); writing—review and editing (lead).

## CONFLICT OF INTEREST STATEMENT

Wei Duan and Cuong V. Pham are the inventors of the patent entitled ‛A fluorescence polarisation‐based method for detection of extracellular vesicles using aptamer’. China National Intellectual Property Administration, Provisional Patent #202210738321.6. World Intellectual Property Organisation and European Patent Office, International Application Number: PCT/CN2023/100289.

## Supporting information

Supporting Information
