# Supplementary material for: An aptamer‐guided fluorescence polarisation platform for extracellular vesicle liquid biopsy
Source: J Extracell Vesicles. 2024 Sep 2;13(9):e12502. doi: 10.1002/jev2.12502 (PMC11367152; doi:10.1002/jev2.12502)
Supplement: Supplementary file 1 — Supporting Information [file JEV2-13-e12502-s001.docx]

**An aptamer-guided fluorescence polarization platform for extracellular vesicle liquid biopsy**

Cuong Viet Pham^1,2^, Rocky Chowdhury^1^, Shweta Patel^1^, Satendra Kumar Jaysawal^1^, Yingchu Hou^3^, Huo Xu^4^, Lee Jia^4^_,_ Yu-mei Zhang^1^, Xiaowei Wang^2,5^, Wei Duan^1,*^ and Dongxi Xiang^6,7*^

1. School of Medicine, Deakin University, Waurn Ponds, VIC, 3216, Australia

2. Molecular Imaging and Theranostics Laboratory, Baker Heart and Diabetes Institute, Melbourne, VIC, 3004, Australia

3. Laboratory of Tumor Molecular and Cellular Biology College of Life Sciences, Shaanxi Normal University 620 West Chang’an Avenue, Xi’an, Shaanxi, 710119, China

4. College of Materials and Chemical Engineering, Minjiang University, Fuzhou, Fujian, 350108, China

5. Department of Cardiometabolic Health, University of Melbourne, VIC, 3052, Australia

6. State Key Laboratory of Systems Medicine for Cancer, Shanghai Cancer Institute, Shanghai Jiaotong University, Shanghai, 201210, China

7. Department of Biliary-Pancreatic Surgery, the Renji Hospital Affiliated to Shanghai Jiaotong University School of Medicine, Shanghai, 201211, China

1. **Materials and methods**
2. **Characterizations of extracellular vesicles**
   1. **Nanoparticle tracking analysis (NTA)**

To determine vesicle counts, samples were treated with either 0.075% Triton X-100 for serum samples or 0.5% Triton X-100 for cell culture -derived EVs in ice water for 20 min to lyse the vesicles. Thereafter, the entire lysed EV samples as well as the respective EVs without Triton X-100 treatment were brought to room temperature for 5 mins before being subjected to nanoparticle tracking analysis (NTA). NTA was performed using a NanoSight NS300 (Malvern) equipped with a 405 nm laser. Three 60-s videos were recorded for each sample with camera level set at 16 and detection threshold set at 11. Temperature was monitored throughout the measurements. Videos recorded were analysed with NTA software version 3.2 to determine the concentration and size distribution of measured particles with corresponding standard error. For analysis, auto settings were used for blur, minimum track length and minimum expected particle size. EV samples were diluted with F-PBS as to specifically fit the optimal working range (20 particles per frame) of the instrument.

- 1. **Scanning electron microscopy (SEM)**

Extracellular vesicles isolated with ultracentrifugation were fixed with 2.5% glutaraldehyde in MilliQ water for 10 min at room temperature. Then, fixed EV solution was attached and dried on the copper grid (Electron Microscopy Sciences, Cat No.: HC300-Cu) in the fume hood for 24 h. Next, dried EV sample was sputtered with a 15 nm conductive gold layer (Leica EM ACE600) to prevent charging EV sample, reduce microscope beam damage, and improve the secondary electron signal before being kept in vacuum chamber prior to image acquisition. Finally, the SEM images of EVs captured using a Zeiss Supra 55 VP scanning electron microscope at 5 kV acceleration voltage.

- 1. **Immunoblotting assay**

Briefly, 16 µL of cell or EV lysate (10 ‒ 40 µg of total proteins) was mixed with 8 µL of 3× loading buffer (33.3% (v/v) glycerol, 6% (v/v) SDS, 0.06% (w/v) bromophenol blue, and 16% (v/v) β-mercaptoethanol in 1M Tris-HCL pH 6.8) in a 1.5-mL Eppendorf tube. The sample was heated to 95 °C for 5 min, then cooled down in ice for 5 min, before being quick centrifugation. Afterward denatured proteins in cell or EV lysate were separated by a 10% SDS-PAGE gel or a 4-15% precast protein gel (Bio-rad, #4561084) under a condition of 250 V and a current of 30 mA/gel. Next, proteins in the gel were electrotransferred onto a nitrocellulose membrane (GE Healthcare Life Sciences, #10600002) or a polyvinylidene difluoride membrane (Merck, #IPVH00010) under a current of 300 mA in 1 h at 4 °C. Following 1-h blocking with 5% skim milk in 1X TBST (0.8766% (w/v) of NaCl, 0.2422% (w/v) of Tris base, 0.1% (v/v) of Tween 20 in milliQ water), the blot was incubated with primary antibodies in 5% skim milk/TBST overnight at 4 °C on a roller mixer (Ratek, Model No.: BTR5). Primary antibodies used were mouse anti-human CD63 antibody (BioLegend, #353039, 1:1000), mouse anti-human Alix antibody (BioLegend, #634501, 1:500), rabbit anti- Calnexin antibody (Santa Cruz, #sc-11397, 1:1000), mouse anti-human EpCAM antibody (BioLegend, #324202, 1:1000), and mouse anti-human HER2 antibody (OriGene, #TA503443BM, 1:000). Primary antibodies, then, were detected using goat anti-mouse IgG antibody (Thermo Scientific, #31430, 1:3000) or goat anti-rabbit IgG antibody (Cell Signaling, #7074, 1:2000) and visualized using the Super Signal West Dura Substrate (Thermo Scientific, #34075). Imaging was performed on ImageQuant LAS-4000 chemiluminescence & fluorescence imaging system (GE Healthcare Life Sciences, USA) or Syngene G:BOX Gel Documentation System (Synoptics, USA).

- 1. **Profiling extracellular vesicle membrane proteins by beads-based flow cytometry**

Firstly, the streptavidin-coated 10-µm magnetic beads (Merck, Cat No.: LSKMAGT02) in original vial were vortexed gently for 30 seconds, then one volume of the beads was washed with 10 volumes of 5% F-BSA, followed by blocking with 10 volumes of 5% F-BSA on a HulaMixer Sample Mixer (Thermo Fisher Scientific, Cat No.: 15920D) for 1 h at room temperature. The supernatant was discarded, and the blocked beads were re-suspended in 1 volume of 5% F-BSA for further isolation of EVs.

Biotinylated EV-capture antibodies and the streptavidin-coated magnetic beads were mixed at a ratio of 0.5 µg of antibody per 1 µL of blocked beads (2.5 µg of dry beads) and incubated on the HulaMixer Sample Mixer or 30 min at room temperature. Then, 2 µL of antibody-coated beads were washed with an excess of 0.22-µm-membrane-filtered PBS plus 0.1% Tween 20 (Washing buffer-B) for three times. Such beads, thereafter, were incubated with a mixture of 100 µL of 5 ×10^8^ ultrafiltrated EVs and 100 µL of 5% F-BSA on the HulaMixer Sample Mixer overnight in a cold room. Then, the EVs immobilized on the beads were washed with an excess of Washing buffer-A for three times before being incubated with 100 µL of 50 nM each of the following antibodies in 5% F-BSA, including APC- or PE-conjugated anti-human CD9 antibody (capture antibodies: anti-CD63/CD81 antibodies), APC- or FITC-conjugated anti-human CD63 (capture antibodies: anti-CD9/CD81 antibodies), APC- or FITC-conjugated anti-human CD81 (capture antibodies: anti-CD9/CD63 antibodies), Alexa Fluor647®-conjugated anti-human EpCAM (capture antibodies: anti-CD9/CD63/CD81 antibodies), or PE-conjugated anti-human HER2 antibody (capture antibodies: anti-CD9/CD63/CD81 antibodies) on a HulaMixer Sample Mixer (Thermo Fisher Scientific, Cat No: 15920D) for 30 min at room temperature. Then, such EV-coated beads were washed with an excess of Washing buffer-B for three times before subjecting to flow cytometric analysis of 10,000 events per sample. The median fluorescence intensity (MFI) and fluorescence histograms of the binding were recorded by BD FACS-Canto™ II flow cytometer and analysed using FlowJo^TM^ (v10.6.2).

1. **Folding of aptamers**
   1. **Folding of CD63-BP aptamer**

Prior to conducting experiments using CD63-BP, the aptamer was diluted by PBS plus 1.0 mM MgCl_2_ to desired concentrations. Then, aptamer solution was denatured at 95 °C for 5 min, followed by incubation in ice for 10 min and re-folding at 37 °C for 15 min.

- 1. **Folding of HER2-HApt aptamer**

Prior to conducting experiments using HER2-HApt aptamer, the aptamer was diluted by PBS plus 5.0 mM MgCl_2_ to desired concentrations. Then, aptamer solution was denatured at 95 °C for 5 min, followed by incubation in ice for 15 min and re-folding at 37 °C for 15 min.

- 1. **Folding of negative control aptamer**

Prior to conducting experiments using control aptamer, the aptamer was diluted by PBS plus 1.0 mM MgCl_2_ to desired concentrations. Then, aptamer solution was denatured at 95 °C for 5 min, followed by incubation in ice for 10 min and re-folding at 37 °C for 15 min.

1. **Determination of dissociation constant of antibody and aptamer on immobilized EVs**

Wherever possible, at least five data points were used in the assays for *K_D_*, a data point with the concentration of the ligand equals to *K_D_*, a data point with the concentration of the ligand 5 times higher or lower than the *K_D_*, and a data point with the concentration of the ligand 10 times higher or lower than the *K_D_* [1]. Briefly, 100 µL of FITC-conjugated anti-human CD63, FITC-conjugated anti-HER2 antibody in F-PBS or folded FITC-labeled CD63-BP aptamer, FITC-labeled HER2-HApt aptamer at different concentrations in 0.22 µm membrane filtered binding buffers were incubated with each well containing 4.0 × 10^8^ HT-29 EVs immobilized with anti-human EpCAM antibody, or 2.0 × 10^9^ SKBR3 EVs immobilized with anti-human CD9/CD81 antibodies for 1 h at room temperature in dark. After washing three times with 200 µL of binding buffer, fluorescence intensity was measured using CLARIOstar Plus microplate reader (BMG Labtech). Control wells as the fluorescence background for analysis were prepared using IgG isotype-matched negative control antibody at corresponding concentrations.

1. **Optimization for FluoPADE assays**
   1. **Optimization of biotinylated antibody concentration per well**

Firstly, streptavidin-coated wells were washed with 200 µL of 0.22 µm filtered PBS plus 0.1% BSA and 0.05% Tween 20 (Washing buffer-A) for twice. Then, 100 µL of capture antibody prepared in Washing buffer-A at a concentration in a range of 2, 5, 8, 10, and 15 µg/mL was added into each well. The well, next, was incubated for 2 h at room temperature in a shaker (Thermoline Scientific, Model No.: TL400) at 120 rpm before thoroughly washed with 200 µL of Washing buffer-A twice and 200 µL of F-PBS once. Afterward, the well coated with desired antibody was incubated with 2 × 10^8^ EVs overnight in a cold room before all unbound EVs and impurities were discarded. The well carrying EVs was washed with 200 µL of Washing buffer-B for three times, followed by adding 100 µL in 5% F-BSA of 50 nM FITC-conjugated anti-human CD63 antibody into each well. The well was incubated in the shaker at 120 rpm for 30 min at room temperature in dark, followed by 3 washes with 200 µL of Washing buffer-B each time. Finally, fluorescence intensity of the well in 100 µL of F-PBS was measured using a microplate reader (CLARIOstar Plus, BMG Labtech) installed with FITC filters (excitation: 482 nm, emission: 530 nm). Control wells as the fluorescence background for analysis were prepared in a similar way but using IgG isotype-matched negative control antibody at corresponding concentrations instead of specific EV-capture antibodies.

- 1. **Optimization of biotinylated antibody coating time**

Streptavidin-coated wells were washed with 200 µL of Washing buffer-A for twice. Then, 100 µL of 8 µg/mL biotinylated capture antibody in Washing buffer-A was added into each well. The well, next, was incubated for 0.5, 1 or 1.5 h at room temperature in a shaker at 120 rpm (Thermoline Scientific, Model No.: TL400) before thoroughly washed with 200 µL of Washing buffer-A once and 200 µL of F-PBS twice. Afterward, the well coated with desired antibody was incubated with 2 × 10^8^ EVs overnight in a cold room. Next, the well carrying EVs was washed with 200 µL of Washing buffer-B for three times before adding 100 µL of 50 nM FITC-conjugated anti-human CD63 antibody in 5% F-BSA into each well. The well, was incubated in the shaker for 30 min at 120 rpm at room temperature in dark, followed by 3 washes with 200 µL of Washing buffer-B each time. Finally, fluorescence intensity of the well in 100 µL of F-PBS was measured using a microplate reader (CLARIOstar Plus, BMG Labtech). Control wells as the fluorescence background for analysis were prepared in a similar way but using IgG isotype-matched negative control antibody at corresponding concentrations instead of specific EV-capture antibody.

- 1. **Optimization of EV capture incubation time**

Similarly, streptavidin-coated wells were washed with 200 µL of Washing buffer-A twice. Then, 100 µL of 8 µg/mL biotinylated EV-capture antibody in Washing buffer-A was added into each well. The well was incubated for 30 min at room temperature in a shaker at 120 rpm (Thermoline Scientific, Model No.: TL400) before thoroughly washed with 200 µL of Washing buffer-A once and 200 µL of F-PBS twice. Afterward, the well coated with desired antibody was incubated with 2.0 × 10^8^ EVs for 2, 4 h at room temperature, or 16 h in a cold room. Next, the well carrying EVs was washed with 200 µL of Washing buffer-B for three times before adding 100 µL in 5% F-BSA containing 50 nM FITC-conjugated anti-human CD63 antibody into each well. The well, then, was incubated in the shaker for 30 min at room temperature in dark, followed by 3 washes with 200 µL of Washing buffer-B each time. Finally, fluorescence intensity of the well in 100 µL of F-PBS was measured using a microplate reader (CLARIOstar Plus, BMG Labtech) installed with FITC filters. Control wells as the fluorescence background for analysis were prepared in a similar way but using IgG isotype-matched negative control antibody at corresponding concentrations instead of specific EV-capture antibody.

- 1. **Optimization of ligand concentration for fluorescence polarization assays**

The concentration of FITC-attached aptamers for formal assays were optimized based on the signal-to-noise (S/N) ratios of parallel and perpendicular intensity against ligand concentrations.

For signal samples, the black streptavidin-coated well (Thermo Fisher Scientific, Cat No.: 15503) used in this assay was washed with 200 µL of Washing buffer-A twice, followed by the incubation with either biotinylated anti-EpCAM antibody or a panel of biotinylated anti-CD9/CD81 antibodies (1:1, w/w) for 30 min at room temperature. Following two washes with 200 µL of Washing buffer-A and one wash with F-PBS, 100 µL of FITC-labelled aptamer prepared in serial concentrations of 1, 5, 10, 25, and 50 nM in binding buffers/F-PBS was added in the well. After 1-h incubation, FP values were recorded and expressed in units of milli P, or mP, which is calculated from the measurements of perpendicular and parallel fluorescence intensity values detected relative to the direction of the polarized excitation light.

Control sample (noise determination) simply is the parallel and perpendicular intensity of the well containing the highest number of immobilized EVs in binding buffer or F-PBS used in the formal assays to exclude possible impacts from immobilized EVs themselves to observed FP signals. Control well was prepared in similar way above for signal samples, however the well-capture antibody after washes was incubated with 100 µL of 2.0 × 10^10^ HT-29 EVs/mL for CD63-BP aptamer-based FP assay or 100 µL of 2.0 × 10^10^ SKBR3 EVs/mL for HER2-HApt aptamer-based FP assays. All EVs were captured for 16 h in a cold room. Afterward the well was thoroughly washed with 200 µL of 0.1% F-PBS for three times before 100 µL of respective binding buffer was added into the well to simultaneously measure parallel and perpendicular intensities with other samples above.

The optimal concentration of ligand was the concentration at which the S/N ratio was equal or higher than 30. Moreover, the FP values over corresponding ligand concentrations were also plotted to determine the aptamer concentration at which the FP value started to be stable.

1. **Results**


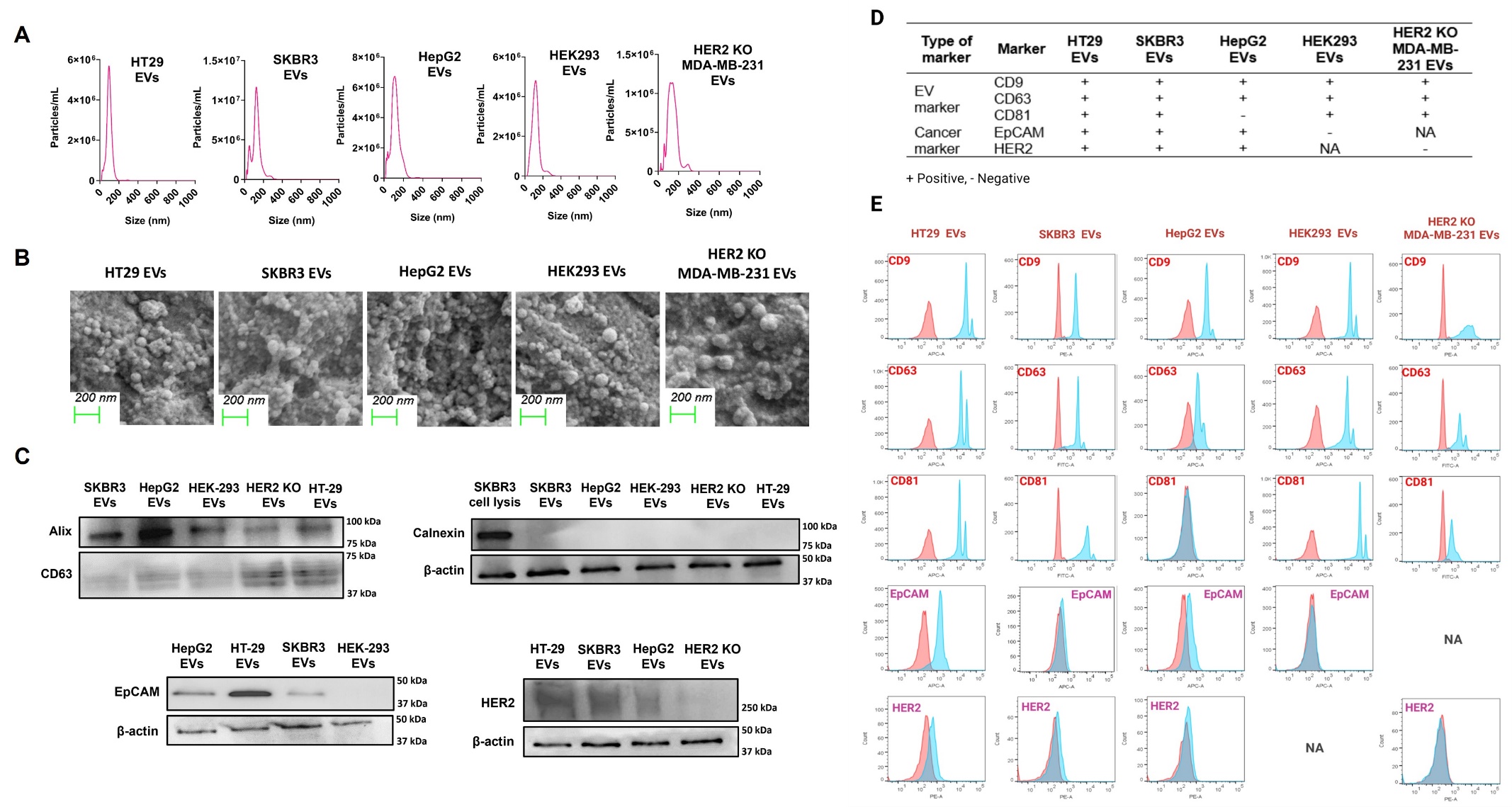


**Figure S1**. **Characterization of EVs from HT-29, SKBR3, HepG2, HEK293 and MDA-MB-231 cells with HER2 gene knocked out** (**A**), Size distribution by nanoparticle tracking analysis. (**B**), Morphology by scanning electron microscope. (**C**), Proteins by Western blot including Alix, CD63, EpCAM, HER2 as well as calnexin. (**D**), Summary of marker expressions on EVs as determined by flow cytometry. (**E**), Respective histograms of EV surface proteins. Red, background control; and blue, marker proteins.

**Figure S2**. **The dissociation constants and fluorescence polarization of aptamers and antibodies to immobilized EVs.** Determination of apparent dissociation constant (*K_D_)* of (**A**), FITC-anti-CD63 antibody to immobilized HT-29 EVs and (**B**), FITC-CD63-BP aptamer to immobilized HT-29 EVs. (**C**), Changes in fluorescence polarization using FITC-anti-CD63 antibody or FITC-CD63-BP aptamer (at the concentration of 5 nM, *K_D_*, and 2*K_D_*) on immobilized HT-29 EVs. Determination of apparent *K_D_* of (**D**) FITC-anti-HER2 antibody to immobilized SKBR3 EVs and (**E**) FITC-HER2-HApt aptamer to immobilized SKBR3 EVs. (**F**), Changes in fluorescence polarization of FITC-anti-HER2 antibody or FITC-HER2-HApt aptamer (at the concentration of 5 nM, *K_D_*, and 2*K_D_*) on immobilized SKBR3 EVs. Data shown are means ± S.D., n=3.


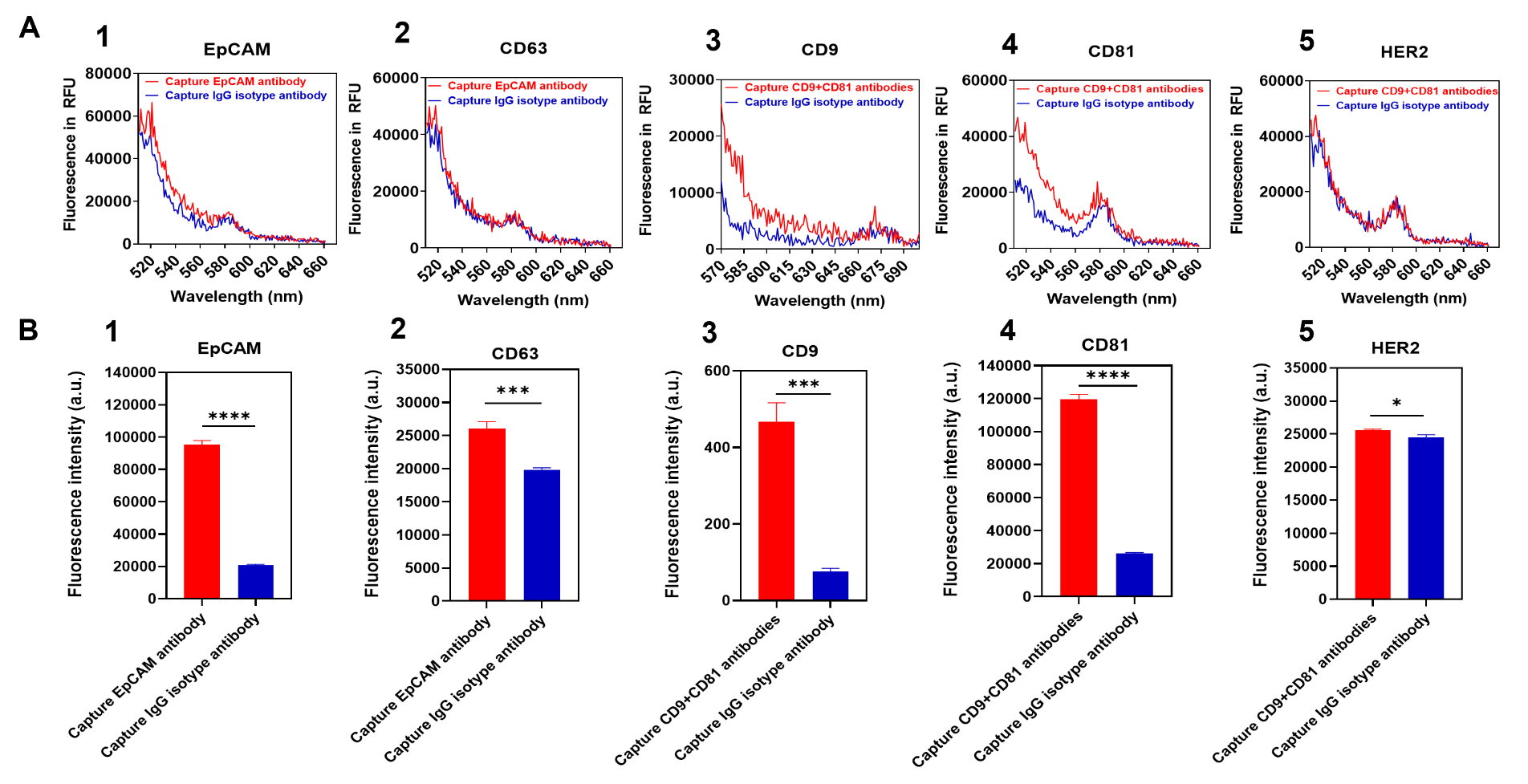


**Figure S3**. **Characterization of EV markers and cancer biomarkers on EVs isolated from human plasma.** EVs from human plasma were immobilized using either anti-EpCAM antibody for determining EpCAM and CD63 on plasma-derived EVs, or anti-CD9/CD81 antibodies for determining CD9, CD81 and HER2 on plasma-derived EVs. The isotype-matched control antibody (IgG) was used as a negative control for EV immobilization. (**A**) The spectra of fluorescence intensity against excitation wavelengths ranging from 520 nm to 660 nm was recorded for FITC-labelled anti-EpCAM antibody (A1), anti-CD63 antibody (A2), anti-CD81 antibody (A4), and anti-HER2 antibody (A5) on EVs. The spectra of fluorescence intensity against excitation wavelengths ranging from 570 nm to 690 nm was also recorded for PE-anti-CD9 antibody (A3). (**B**), The respective fluorescence intensity measured for (B1) anti-EpCAM antibody (ex: 485 nm, em: 535 nm), (B2) anti-CD63 antibody (ex: 485 nm, em: 535 nm), (B3) anti-CD9 antibody (ex: 560 nm, em: 575 nm), (B4) anti-CD81 antibody (ex: 485 nm, em: 535 nm), and (B5) anti-HER2 antibody (ex: 485 nm, em: 535 nm) on immobilized EVs from human plasma. Data shown are means ± S.D., n=3. **P* ≤ 0.05, ****P* ≤ 0.001, and *****P* ≤ 0.0001.


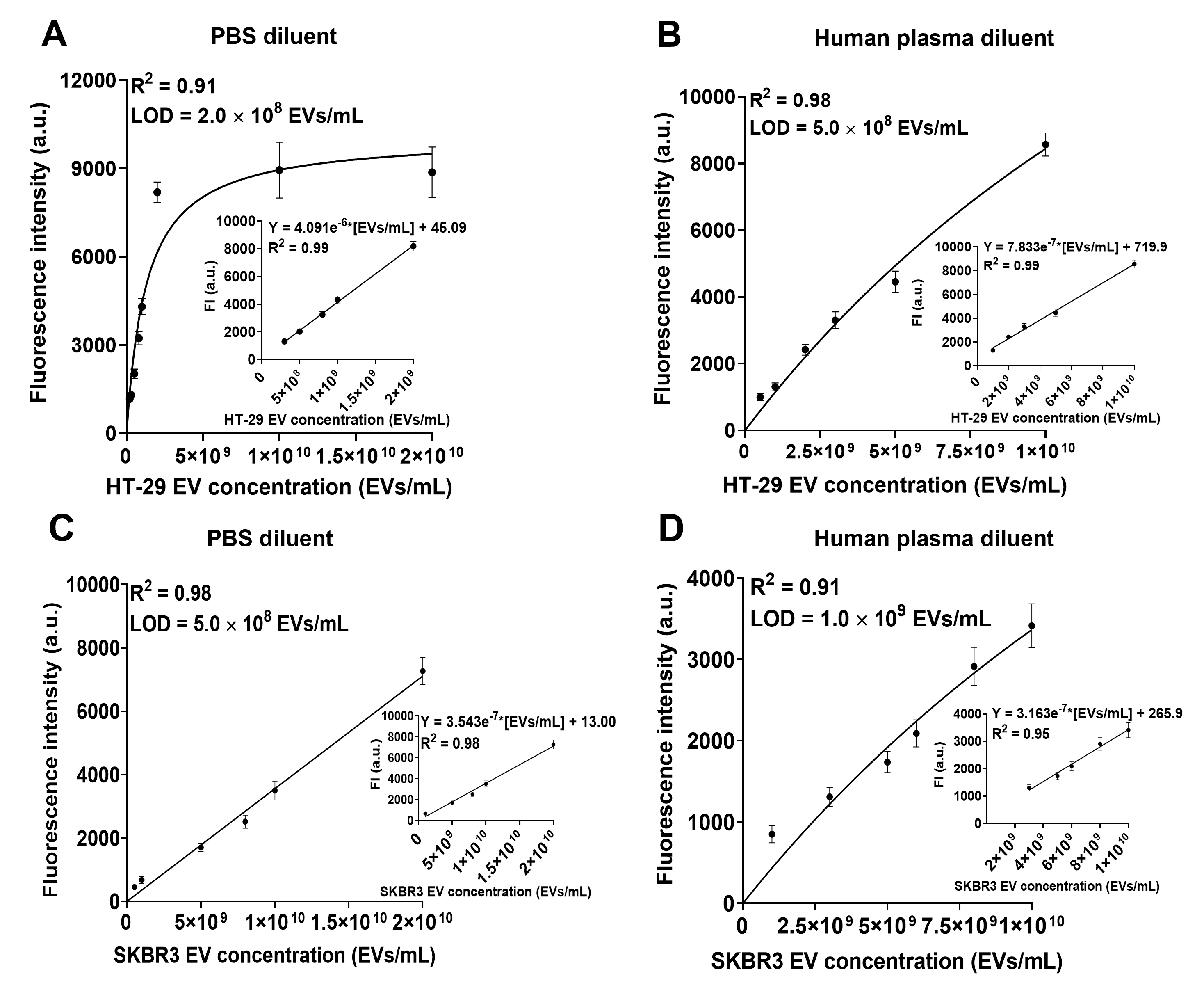


**Figure S4**. **Limit of detection and linear dynamic range of fluorescence intensity-based assays using aptamers**. Fluorescence intensity as a function of EV concentration and linear plots (inset) of fluorescence intensity as a function of EV concentration are presented, along with the corresponding limit of detection and linear dynamic range of fluorescence intensity assays. (**A-B**) FITC-CD63-BP aptamer with EVs from HT-29 cells. (**C-D**) FITC-HER2-HApt aptamer with EVs from SKBR3 cells. Cell line-derived EVs were either suspended in PBS (**A, C**) or diluted in human plasma at a 1:9 ratio (**B, D**). Data shown are means ± S.D., n=3.


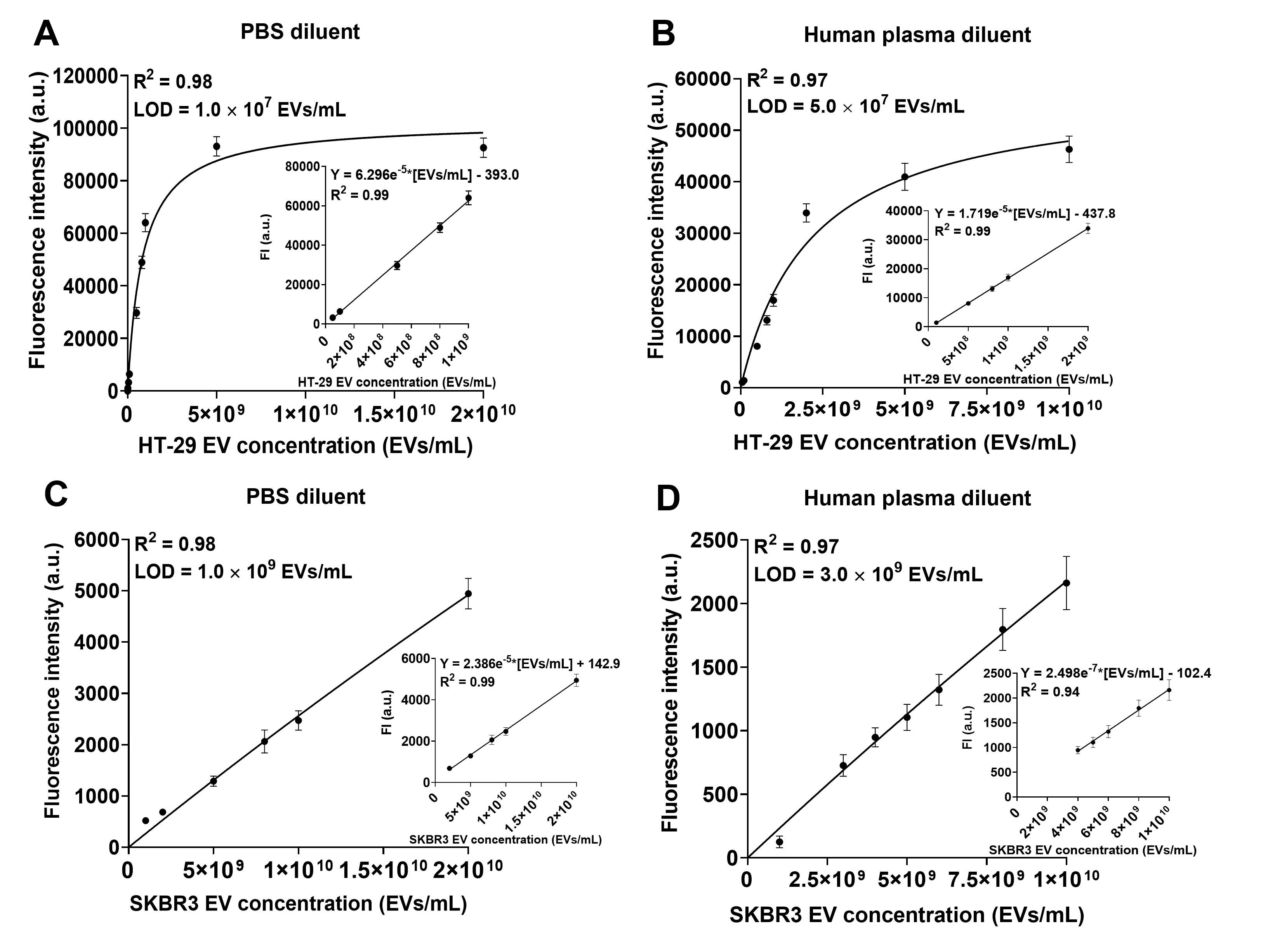


**Figure S5**. **Limit of detection and linear dynamic range of fluorescence intensity-based assays using antibodies** Fluorescence intensity as a function of EV concentration and linear plots (inset) of fluorescence intensity as a function of EV concentration are presented, along with the limit of detection and linear dynamic range of fluorescence intensity assays. (**A-B**) FITC-Anti-CD63 antibody with EVs from HT-29. (**C-D**) FITC-Anti-HER2 antibody with EVs from SKBR3. Cell line-derived EVs were suspended at 1:9 ratio either in PBS (**A, C**) or in human plasma (**B, D**). Data shown are means ± S.D., n=3.


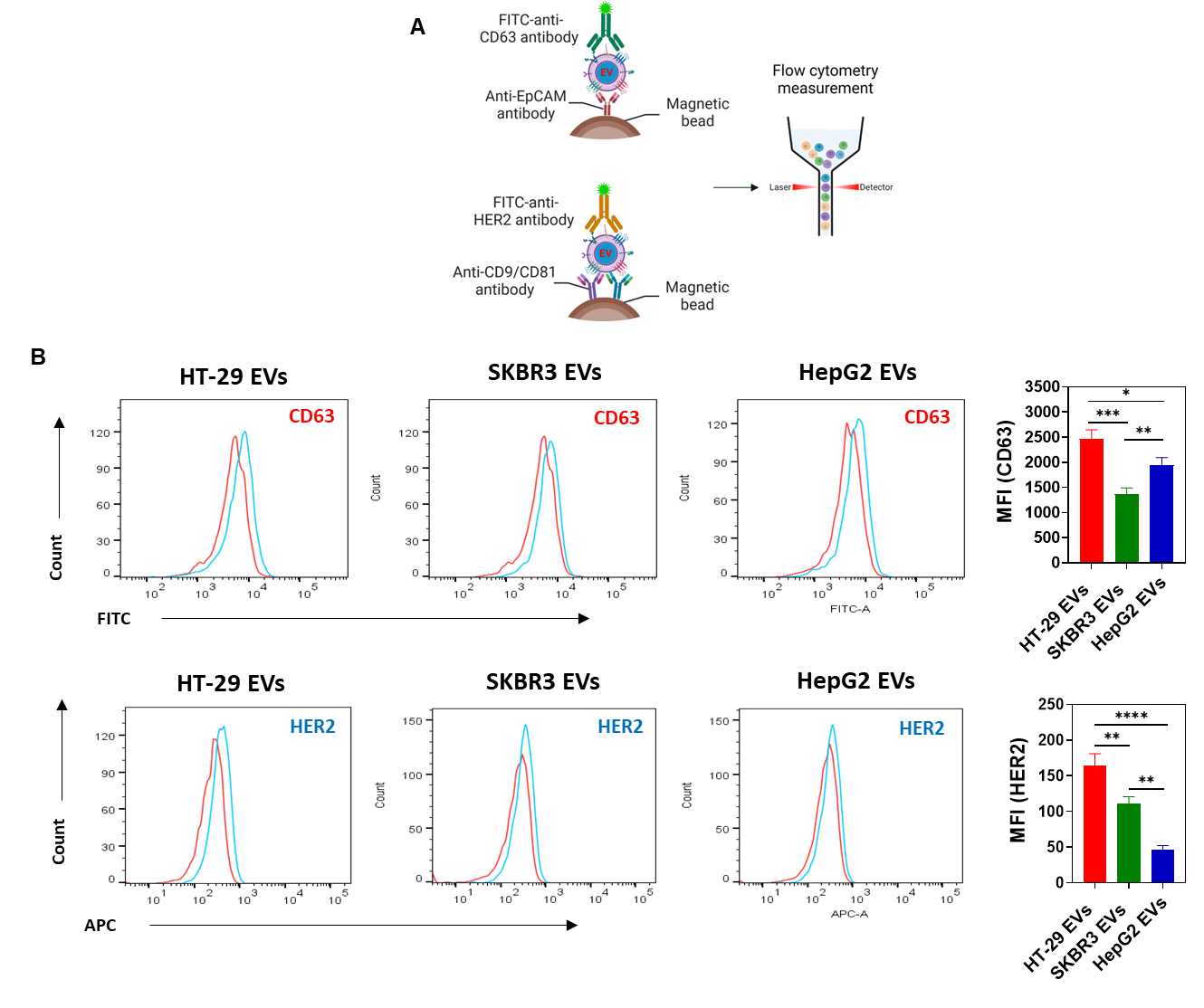


**Figure S6**. **Determination of the level of CD63 and HER2 on the surface of EVs immobilized onto antibody-coated magnetic beads using flow cytometry**. (**A**), Schematic illustration of bead-based flow cytometric assay for EV surface proteins. (**B**), **Top panel**, the expression levels of CD63 on HT-29 EVs, SKBR3 EVs and HepG2 EVs immobilized on anti-EpCAM antibody-coated magnetic beads (10 μm). **Bottom panel**, the expression levels of HER2 on HT-29 EVs, SKBR3 EVs and HepG2 EVs immobilized on anti-CD9/CD81 antibody-coated magnetic beads. Red trace: background fluorescence intensity of EVs incubated with isotype-matched control antibody (IgG)-coated magnetic beads; blue trace: fluorescence intensity of EVs immobilized on either anti-EpCAM-coated magnetic beads (top panel) or anti-CD9/CD81 antibody-coated magnetic beads (bottom panel). Data shown are means ± S.D., n=3. **P* ≤ 0.05, ***P* ≤ 0.01, ****P* ≤ 0.001, *****P* ≤ 0.0001.

**Table S1. Stability of FP signal over time**

|  | **ΔFP with 1-h incubation of aptamer (mP)** | **ΔFP with 3-h incubation of aptamer (mP)** |
| --- | --- | --- |
| FluoPADE using CD63-BP | 10.1 ± 0.7 | 10.3 ± 0.5 |
| FluoPADE using HER2-HApt | 8.7 ± 0.4 | 9.0 ± 0.4 |

*5.0 × 10^9^ HT29 EVs and SKBR3 EVs were added in the well.*

**References**

1. Goodrich, J.A. and J.F. Kugel, *Binding and kinetics for molecular biologists*. 2006: Cold Spring Harbor Laboratory Press. x, 182 pages.

**Appendix: Full Western blots from which Figure S1C was constructed.**

**
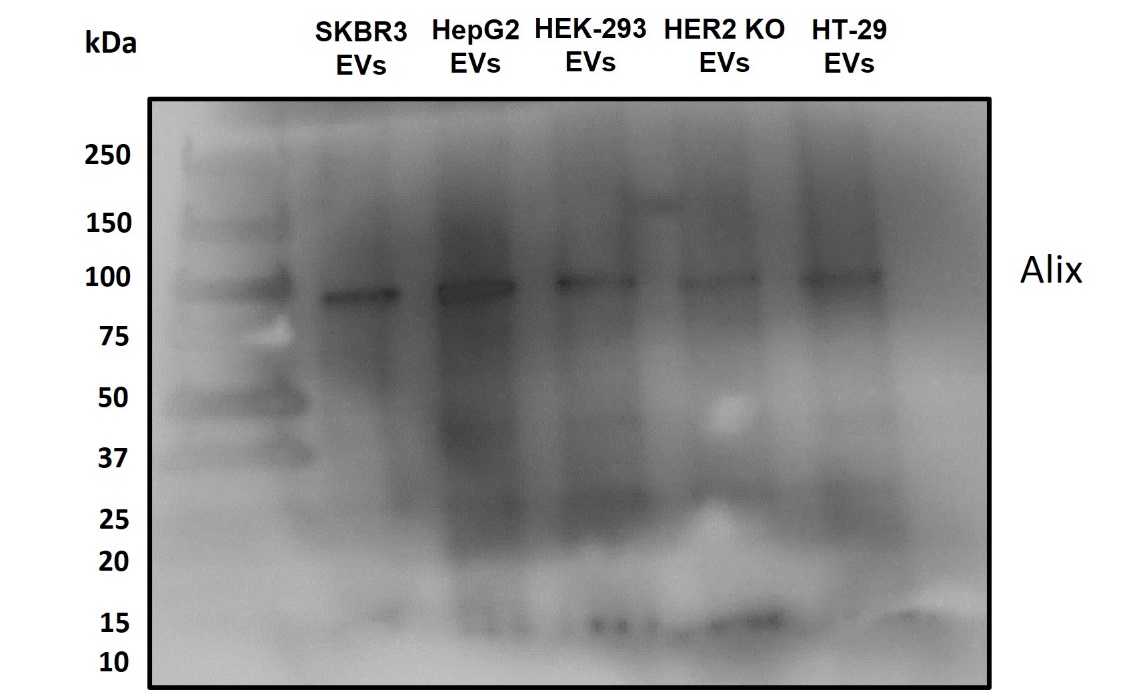
**

**
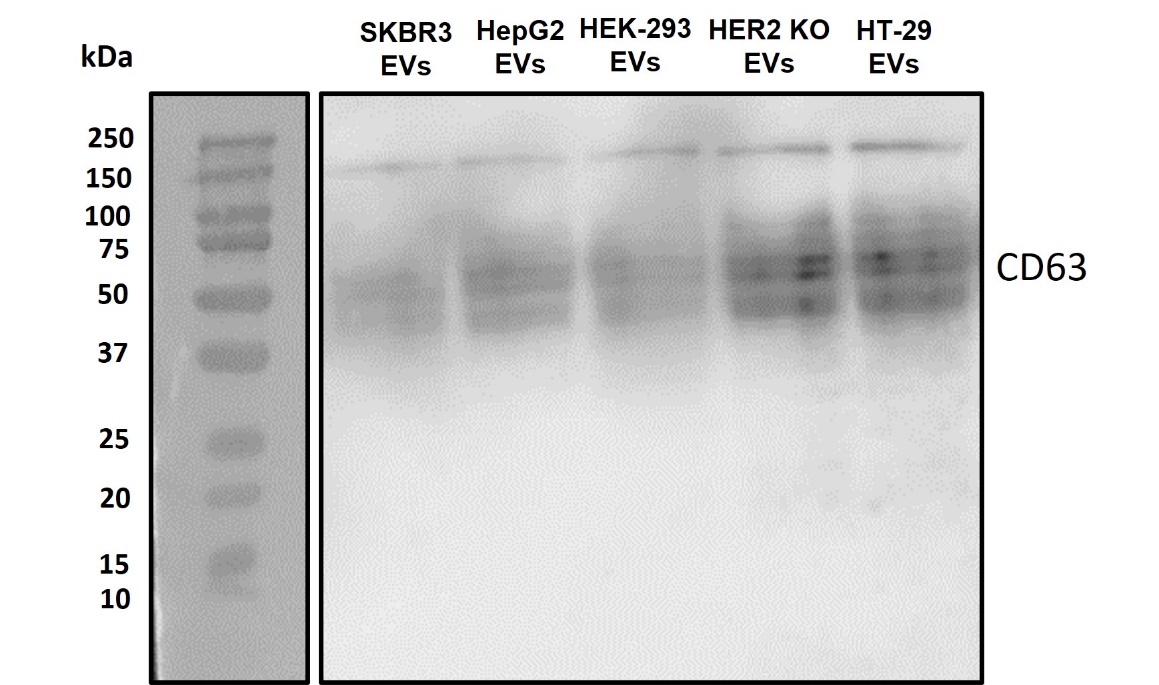
**

**
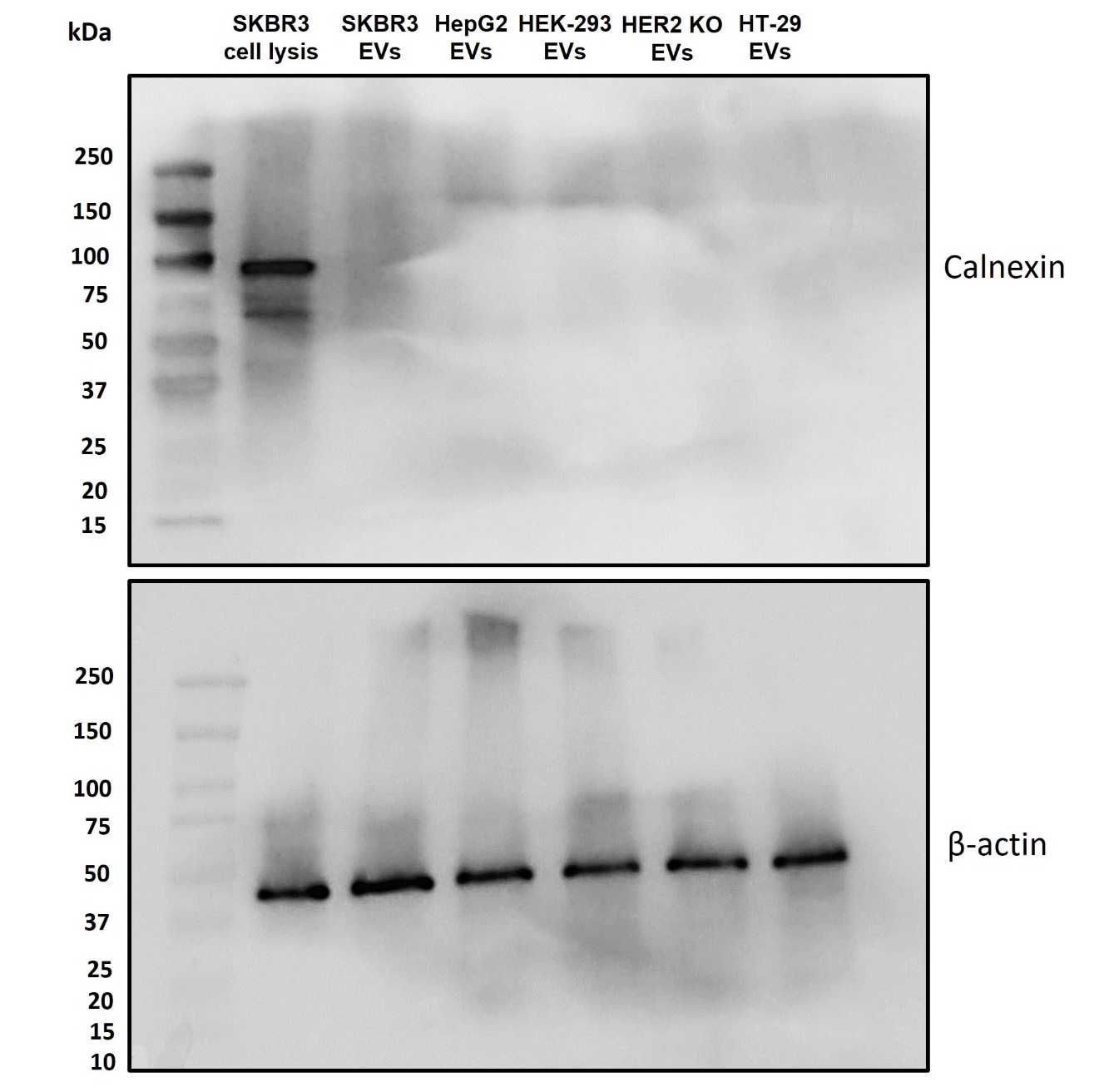
**

**
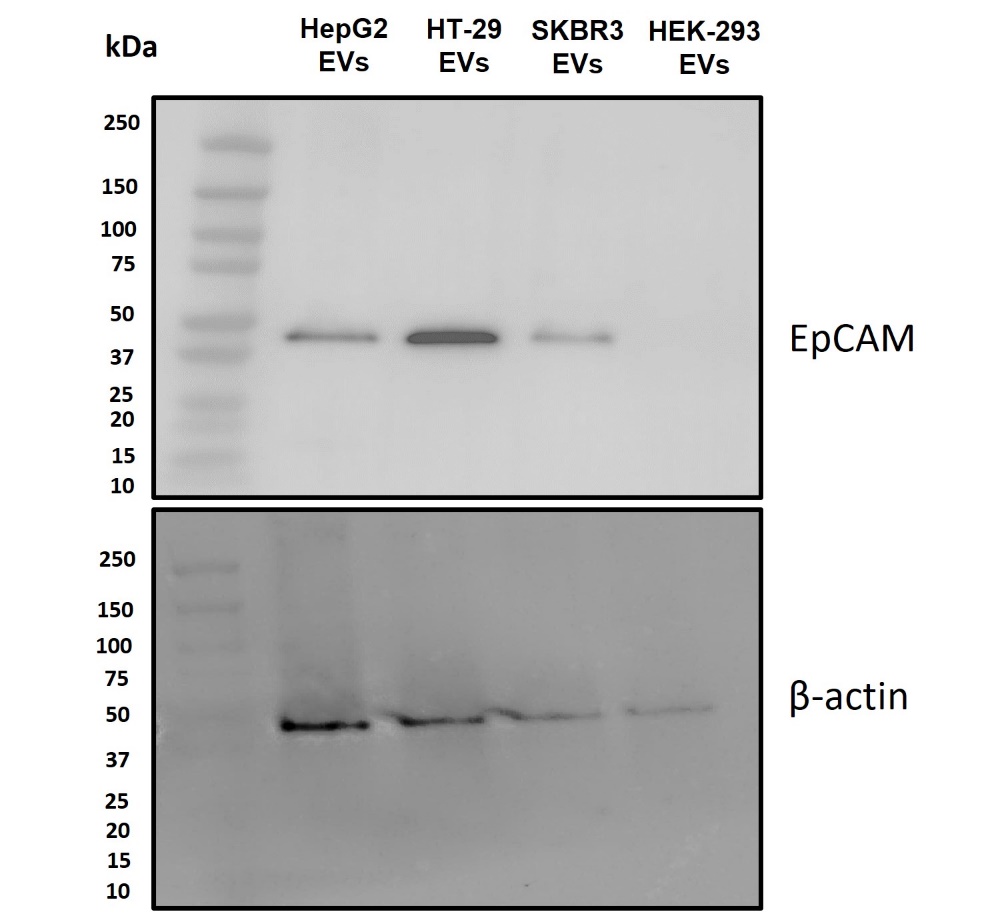
**

**
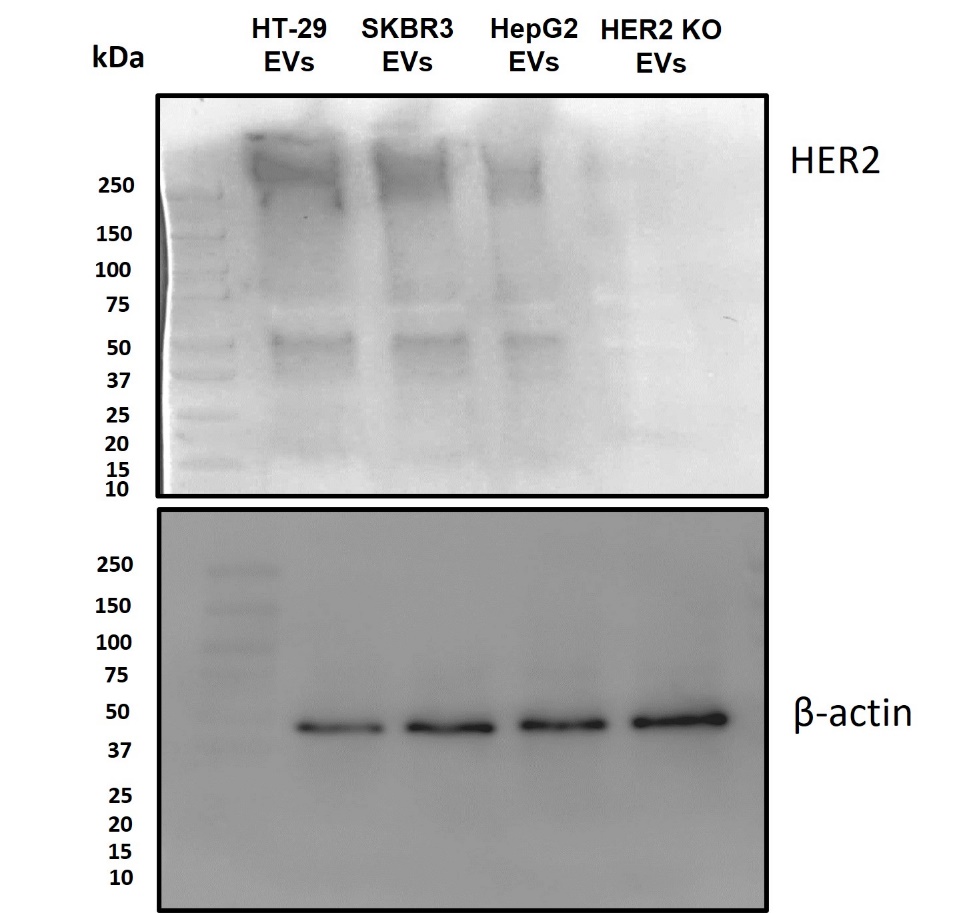
**
